# Unsupervised Scalable Statistical Method for Identifying Influential Users in Online Social Networks

**DOI:** 10.1038/s41598-018-24874-2

**Published:** 2018-05-03

**Authors:** A. Azcorra, L. F. Chiroque, R. Cuevas, A. Fernández Anta, H. Laniado, R. E. Lillo, J. Romo, C. Sguera

**Affiliations:** 1 Universidad Carlos III de Madrid, Leganés, Madrid, Spain; 2 IMDEA Networks Institute, Leganés, Madrid, Spain; 3 Department of Mathematical Sciences, Universidad EAFIT, Medellín, Colombia; 4 Department of Statistics, Universidad Carlos III de Madrid, Madrid, Spain; 5 UC3M-BS Institute of Financial Big Data, Universidad Carlos III de Madrid, Madrid, Spain

**Keywords:** Applied mathematics, Computational science, Computer science, Statistics

## Abstract

Billions of users interact intensively every day via Online Social Networks (OSNs) such as Facebook, Twitter, or Google+. This makes OSNs an invaluable source of information, and channel of actuation, for sectors like advertising, marketing, or politics. To get the most of OSNs, analysts need to identify influential users that can be leveraged for promoting products, distributing messages, or improving the image of companies. In this report we propose a new unsupervised method, Massive Unsupervised Outlier Detection (MUOD), based on outliers detection, for providing support in the identification of influential users. MUOD is scalable, and can hence be used in large OSNs. Moreover, it labels the outliers as of shape, magnitude, or amplitude, depending of their features. This allows classifying the outlier users in multiple different classes, which are likely to include different types of influential users. Applying MUOD to a subset of roughly 400 million Google+ users, it has allowed identifying and discriminating automatically sets of outlier users, which present features associated to different definitions of influential users, like capacity to attract engagement, capacity to attract a large number of followers, or high infection capacity.

## Introduction

Online Social Networks (OSNs) such as Facebook, Twitter, or Google+ have rapidly become the most used online services, through which billions of users intensively interact every day^1^. This makes OSNs an invaluable resource for sectors like advertising, marketing, or politics, which can use them for collecting information and launching campaigns. A challenging important problem is the identification of influential OSNs users, which can be leveraged by the abovementioned actors for, e.g., advertising a product, propagating a message, or improving the image of a company.

The research community has devoted significant effort in characterizing influential OSNs users^2–7^. However, most existing works define a priori the properties that identify influential users, and then use mechanisms based on that definition to find them^4,8–11^. These supervised techniques have two main drawbacks. First, they require considerable manual analysis of the problem and the data for the definition of properties. Second, their effectiveness is fully tied to the definition: if such definition is inaccurate or unsuitable in a given context, the results would be likewise inaccurate or unsuitable. Therefore, effective unsupervised methods to assist in the detection of influential users would be greatly advantageous. Recently proposed methods for outlier detection in the area of functional data analysis (henceforth FDA)^12^ could be applied to this problem as a form to identify different classes of outliers, which are likely to meet the requirements of different influential user’s definitions. Unfortunately, their outlier detection performances are poor with respect to MUOD, or their computational efficiency does not allow applying them to current OSNs (billions of users, each, characterized by tens of variables). (See supplementary material for more details).

## Contributions

**Figure 1:**
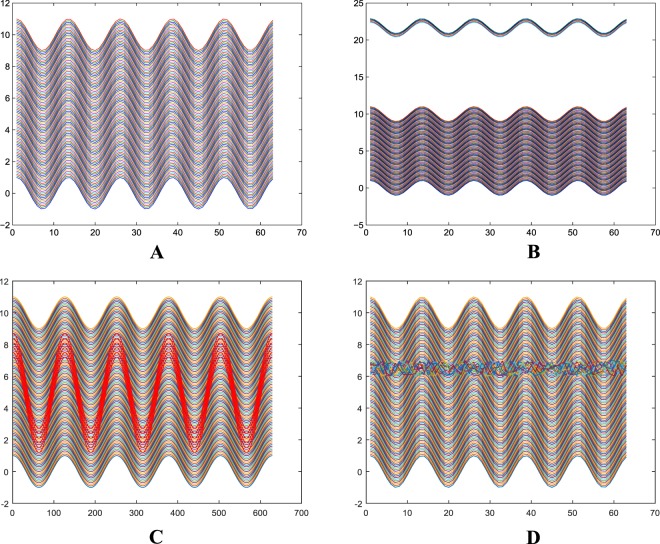
Example of representation of users’ characteristics in the form of a signal (**A**); Example of signals associated to magnitude outliers (**B**); Example of signals associated to amplitude outliers (**C**); Example of signals associated to shape outliers (**D**).

In this report we present a new unsupervised method, that we call Massive Unsupervised Outlier Detection (MOUD), for supporting the identification of influential users in OSNs. MOUD is based on outlier detection in the area of FDA, and it scales to its application in OSNs with millions of users. MUOD considers the characteristics of a user in the form of signal as shown in the example of Fig. 1. Each point in the x-axis is one of the user’s characteristics and the correspondent value for each characteristic is represented in the y-axis. Figure 1. A shows the signal associated to a set of users with similar characteristics. MUOD identifies three types of outliers: (a) Magnitude outliers, whose associated signals present a magnitude significantly different from the mass of users; (b) Amplitude outliers, whose associated signals present an amplitude significantly different from the mass of users; (c) Shape outliers, whose associated signals present a shape significantly different from the mass of users. Figure 1B–D show a graphical example of signals associated to Magnitude, Amplitude and Shape, respectively. Finally, by considering the intersection of these three sets of outliers, MUOD provides a total of 7 differentiated outlier classes.

Our trials with real data sets (see supplementary material) prove that MOUD is as effective in the identification of outliers as the best state-of-the-art methods, while its much higher computational efficiency allows to apply it to much larger scale problems, including the large data scale of current OSNs. We have applied MOUD to a dataset including a complete snapshot of the social graph of Google+ (400 million nodes) as well as the overall public activity of this OSN in its two first years of operation (more than 1 billion interactions). In particular, our goal is to test the ability of MOUD as a support algorithm in the identification of influential users without pre-defining a target profile. The obtained results confirm the applicability of our methodology in practice; since it is capable of finding separate sets of outliers that include different types of influential users based on their capacity to generate engagement, attract followers or their infection capabilities, in the considered large-scale OSN. Hence our proposed method offers unsupervised support to identifying influential users in OSN in those cases where there is not a predefined type of outlier. In turn, as described later, MOUD opens alternative paths for the exploration of interesting entities in other online systems, like Social Media or Online Advertising. Additionally, MOUD could also be applied to the identification of relevant nodes in big data problems from other disciplines (neuroscience, immune interactions, …).

### Identification of Influential Users in OSNs

Users in OSNs can be profiled by a set of parameters that quantify their connectivity, activity, and other relevant characteristics:

- **Connectivity parameters** include: in- and out-node degree (friendship or follower relationships), different centrality metrics, clustering coefficient, and others.
- **Activity parameters** are usually divided into two groups. One group covers the actions directly performed by a user. These usually include published posts and *likes* (*plusones* in Google+) on other users’ posts. The other group includes the reactions that a user’s post generates, including likes from other users, comments from other users, and reshares or reposts (i.e., retweets in the case of Twitter).
- Each user has some **profiling data** in an OSN. The information available in the profile varies from one OSN to another, but typical information relates to the user’s name, location (e.g., city where she lives), job, education, gender, and related data.

Parallel coordinates allow representing users as real functions, and adapt FDA techniques to this problem. Thus, outliers’ observations detected in a functional data set are likely to relate to different definitions of influence in Online Social Networks, such as the capacity of creating engagement among other users, the capacity of attracting a large number of followers, or the capacity of spreading messages to a large number of other users. The first goal of MUOD is identifying outlier users that present significant differences from the mass of users. In FDA, an outlier is defined as an observation generated by a functional random variable with a distribution that is different from the one generating the observations of a functional sample^13^.

Hubert *et al*.^12^ set up a taxonomy of functional outliers that any procedure should detect: shift/magnitude outliers, which have the same shape of the majority, but are shifted away; amplitude outliers, curves that may have the same shape as the majority but their scales differ; and shape outliers, curves whose shape differs from the majority. These qualitative definitions can be a useful tool to isolate users with different behavior but without making any a priori assumption about them. In the literature of FDA, there are recent methods that search for such outliers; however we have found that many of them are not able to distinguish between different types of outliers, nor are computationally efficient when dealing with a large number of observations (see supplementary material). These facts motivate the new outlier detection method that we have developed here. The main contribution of this work is a scalable procedure that can identify the three types of outliers separately. MUOD assigns three values to each OSN user, which defines how different the user is from the mass of users in shape, magnitude, and amplitude (note that a user can be an outlier of several types). The calculation of the three values for a given user is based on the different elements that appear when a linear regression (correlation coefficient, constant, and slope) is fitted between two curves evaluated in the same finite number of points. The larger a value, the “more outlier” the user is. In the supplementary material we have shown that MUOD, when compared with outlier detection approaches recently introduced in the literature, is always among the best options in presence of any type of outlier and thanks to its scalability does not entail the important computational limitations of its best competitors.

**Figure 2:**
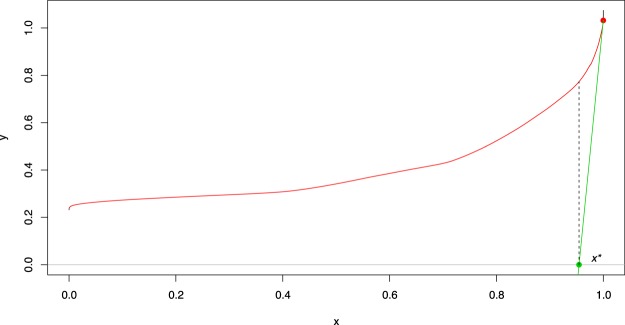
Illustration of the criterion to determine which users are flagged as shape outliers by MUOD. The horizontal x axis represents sample percentiles based on the shape index. The vertical y axis represents shape index values.

Hence, outliers are the users with a “relatively high” value in the shape, magnitude or amplitude value. The second step in our methodology is to establish an appropriate criterion to determine which users are considered outliers, and which users are considered as part of the mass. In MUOD, we have selected the technique proposed by Louail *et al*.^14^. The approach is to first sort the users from the lowest to the highest value in the corresponding variable (e.g., shape, see Fig. 2). Then, we can adjust a continuous monotonically non-decreasing curve from the sorted values. Finally, we find the point x* in which the line tangent to the rightmost point of that curve intersects the x-axis. A user whose position is at least x* is considered outlier of that type (shape in our case).

Once the outliers of the three types have been obtained, in order to classify them further, we split them according to the intersection of the three sets. Thus, we obtain 7 different subsets of different users: 1 group of users outliers of the three types simultaneously, 3 groups of users outliers of exactly two types, and 3 groups of users outliers of only one type. Therefore, MUOD automatically identifies outlier users, and classifies them in 7 different classes. As discussed above, these users are likely to be influential users. We will illustrate this in the next section, where we apply our method to Google+, one of the most popular OSNs in the current Social Media market.

### Testing MUOD in Google+

Google+ was released in June 2011 and, with the support of Google accounts, it has officially more than 2.5 billion registered users. This would make Google+ the largest OSN in number of users, followed by Facebook and Sina Weibo (a OSN that operates in China). In a previous study^15^, we collected a large-scale dataset including: (i) the connectivity graph of the Largest Connected Component of Google+, including around 400 million users; and (ii) the public activity registered in Google+ during a period of two years, since its release in June 2011 until July 2013, accounting with a total of 541 million posts, 1 billion likes, 140 million reshares, and 408 million comments. At the time of the dataset collection, Google+ had a reportedly number of registered users over half a billion.

We leverage this dataset to validate the performance of the proposed method in pre-filtering users in different outlier classes, which likely include users meeting the criteria of different definition of influence within Google+. To this end, we consider n = 21 parameters for each user, covering connectivity, activity, and user profile information. For instance, parameters related to the influence of a user are:

- Number of friends and followers: characterize the popularity of a user
- Number of published posts: characterizes the level of activity of a user in the network
- Number of received likes (plusones), reshares, and comments to the users’ posts: characterize the influence capacity of a user to create engagement.
- Pagerank: characterizes the topological importance of the user in the (unweighted) network.
  The full list of parameters used and their description can be found in the supplementary material.
  As in any OSN, the distribution of the value of activity parameters (e.g., number of published posts) is heavily skewed^16,17^. This implies that there is a huge portion of the population that presents almost no measurable activity (i.e., they typically consume posts published by others but never publish their own posts nor react to posts made by others). Therefore, we have pre-processed the data to remove users of low interest. To this end, we have removed all users with less than 10 public posts in our 2 years activity dataset, since they do not have a substantial and sustained activity.
  After applying this filtering, we obtain a dataset of 5,619,786 users. The application of the method described in the previous section to them yields a total of 302,345, 6,178, and 6,103 outliers of shape, amplitude, and magnitude, respectively. However, the set of shape outliers completely contains the other two sets. Hence, MUOD produces in this case four different sets of outliers (see Fig. 2):
- A set MAS (mag + amp + sha) of 4,036 outliers of the three types simultaneously.
- A set MS (mag + sha) of 2,067 outliers of magnitude and shape simultaneously.
- A set AS (amp + sha) of 2,142 outliers of amplitude and shape simultaneously.
- A set SHA (sha) of 294,100 outliers of only shape.

**Figure 3:**
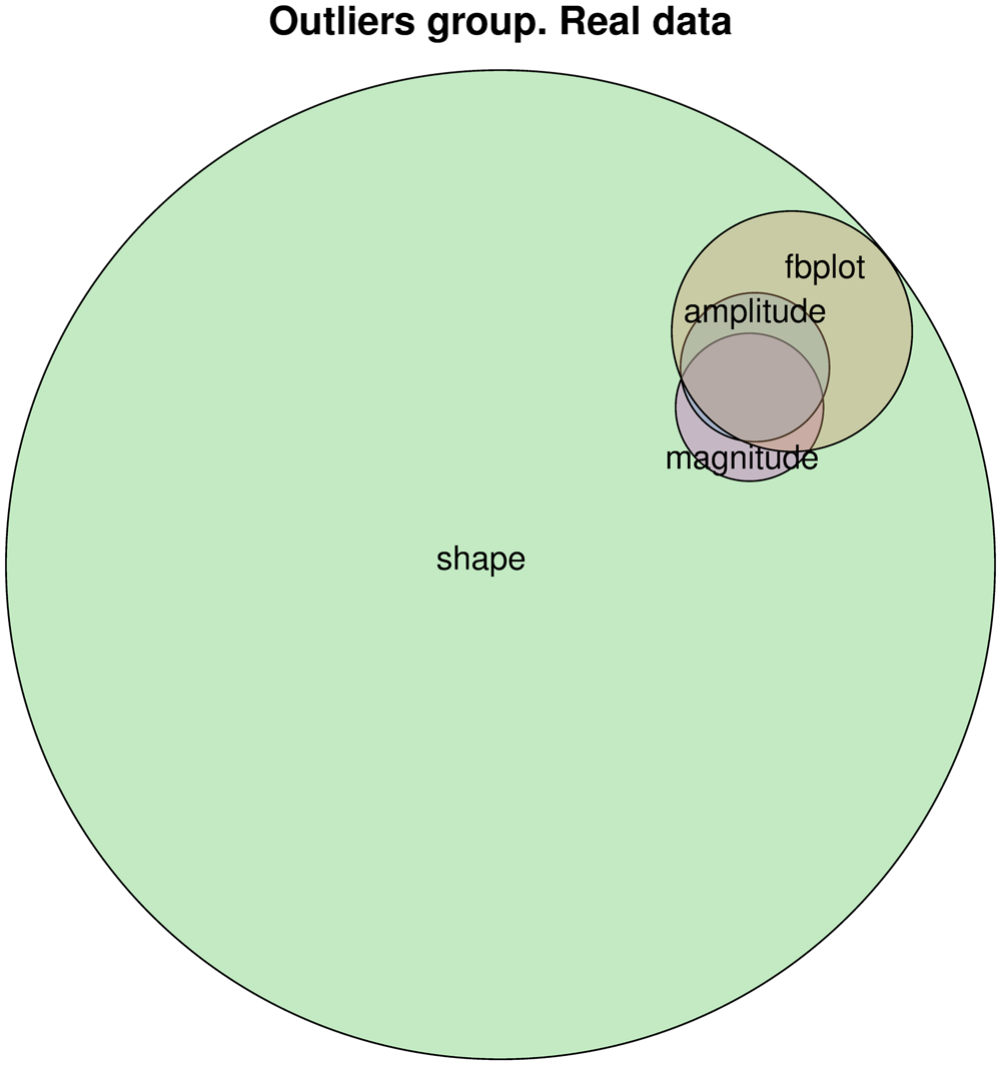
Venn’s diagram that describes the relationship between the different sets of outliers identified by MUOD (and the FBPLOT algorithm).

**Figure 4:**
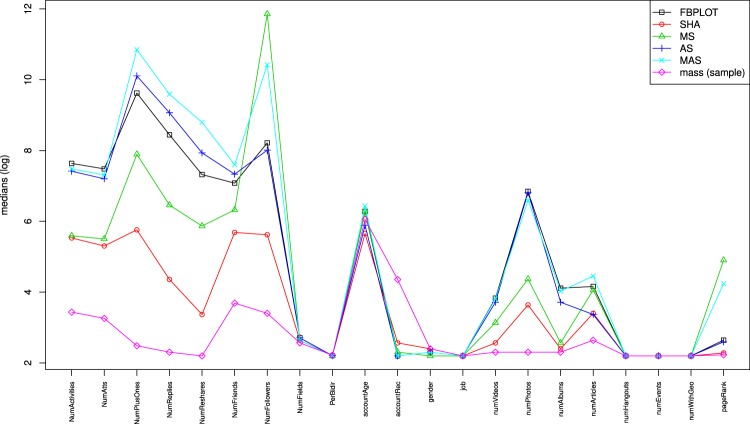
FBPLOT outliers, MUOD outliers (four types) and sample of non-outlying users: parallel coordinates representation of their (log) medians.

**Figure 5:**
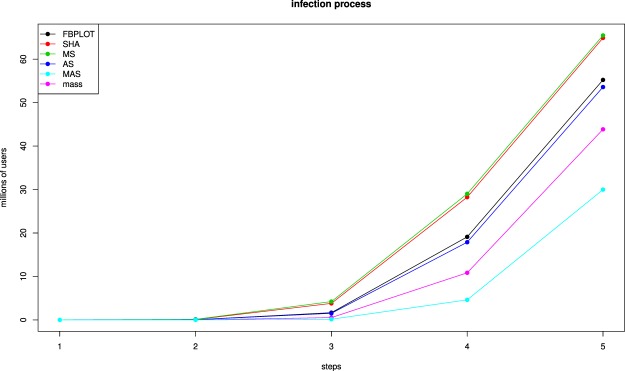
Disease propagation simulations for the different outlier classes. Each line is the result of 10 SI (susceptible-infected) simulations using the centroid user/node of each outlier class as infection root. The simulations were carried out using the largest connected component of the network of followers (around 170 M nodes) and an infection rate of 0.2.

Out of the methods for outlier detection in the current state of the art of FDA only one is computationally efficient to be able to process the millions of users we consider: FBPLOT^18,19^. This method extends the standard boxplot to the FDA framework and allows the identification of outliers. However, as shown in the supplementary material, for FBPLOT it is hard to detect correctly amplitude and shape outliers. In Figs 3–5 we also report the results of this method, while in Fig. S16 in the supplementary material we show results about a synthetic large data set.

The first observation to be made is that each of these sets is qualitatively different from the others, and from the “mass” (i.e., the users not identified as outliers, see Fig. 4). For instance, it can be seen that while the users in the mass have low values in all the parameters, the users in the above 4 outlier classes have relatively higher values in some of them. Moreover, we also observe significant differences across the different outlier classes (note that the y-axis represents log scale). If we consider the influence issue, we observe some of the identified outlier classes seem to meet the requirements of different influence concepts. For instance, we can consider influencers those users able to create engagement among other users through different types of reactions^6,7^ (likes/plusones, replies and reshares). Figure 4 shows that all the outlier classes identified, and in particular MAS and AS, present a volume of reactions (and hence engagement) that is orders of magnitude larger than the one of regular users (represented by the mass). Similarly, if we consider as influencers those users having a large number of followers (or higherdegree)^10,20,21^, the MAS and MS classes show orders of magnitude more followers than the users in the mass, suggesting that this group may include influencers meeting this criterion. Finally, we may consider influential users those able to propagate messages to a larger number of other users emulating an infection process^3–5^. Then, Fig. 5 shows that the MS and SHA outlier classes derived from our Google+ dataset have a significantly higher infection capacity than regular users from the mass.

FBPLOT identifies 16,140 outliers, all of them contained in the set of shape outliers, and with non-empty intersection with the other sets. The outliers identified by FBPLOT are in fact different from the mass, and seem to have properties similar to the users in AS (see Figs 4 and 5). However, simply comparing the relative sizes, one can conclude that FBPLOT leaves out many users that MUOD has detected as outliers (especially of shape). This can be seen clearly when comparing the sets of users that belong to SHA and are included (9,618) or not (284,482) in the set of FBPLOT outliers. The qualitative difference between these users is very small, and the reason for not being considered outliers by FBPLOT is rather weak.

## Conclusions

In this paper, we have introduced MUOD, a novel unsupervised outlier detection algorithm based on FDA theory. MUOD outperforms other FDA-based outlier detection algorithms while offering a high scalability that allows to apply it in large scale multivariable datasets.

We have tested the practical utility of MUOD in a specific problem, the detection of influencers in OSNs. The application of MUOD in a large-scale Google+ dataset including detailed information for more than 400 M users and billions of activities (posts, likes/plusones, reshares, etc) reveals that the different outlier classes identified by MUOD include users that respond to different definitions of influence previously used in the literature. Hence, the results show strong evidences of the utility of MUOD as an algorithm support the unsupervised identification of influencers when a pre-defined type of influential user does not exist.

MUOD algorithm can be applied to a myriad of problems, in which the nodes/users/entities can be defined by a set of properties mapped into a signal. As future work we will explore the utilization of MUOD to address the following issues:

(i) Fake News detection in Social Media. News can be characterized by a large set of properties including (source of the new, timestamp, geographical origin, topic, number of nodes forwarding the news in the OSN, etc). Our hypothesis is that (at least) some types of Fake News will present specific properties that make them different from the rest. If such hypothesis is correct, MUOD should identify them as a certain types of outliers.
(ii) Fraud Detection in Online Advertising. Online Advertising is a multibillion dollar business that represents the main source of revenue for Internet. One of the main problems that online advertising faces is fraud, e.g., when a robot instead of a human watches an ad. We can create a signal associated to different websites in order to identify whether the ads presented in that website are actually viewed by human beings. This signal would include input data such IP address visiting the website, browsers visiting the website, number of ads served per hour, average time spent by users in the website, average mouse movement pattern, etc. Our hypothesis is that some websites committing ad fraud will present some specific properties that make them different from the mass. If this hypothesis hold, MUOD is a good candidate algorithm to be applied in this context.

Beyond online systems, there are several real scenarios where the number of variables and the number of observations are high in which outlier detection is very important. For example, medical imaging datasets often contain deviant observations due to acquisition or pre-processing artifacts or resulting from large intrinsic inter-subject variability. Specifically in Neuro-imaging, various kinds of acquisition artifacts may be present in fMRI (functional Magnetic Resonance Images) data. Even small movements of the head may produce large artifacts in the signals, and also heartbeat and breathing both induce pulsatile motion in the brain, which creates physiological noise artifacts directly in the data^22–25^. Complexity and massive amount of this kind of data, and the presence of different types of noises, makes the fMRI data analysis a challenging one; that demands robust and computationally efficient statistical analysis methods as MUOD.

High-dimensional data are increasingly encountered in other applications of statistics, e.g. in biological and financial studies^26,27^. Also, in geochemical data, because of their complex nature^28^, regional geochemical datasets practically always contain outliers. In fact, finding data outliers that may be indicative of mineralization (in exploration geochemistry) or contamination (in environmental geochemistry) is one of the major aims of geochemical surveys^29^.

## Electronic supplementary material

Supplementary Material
