# Author Correction: Unsupervised Scalable Statistical Method for Identifying Influential Users in Online Social Networks

**DOI:** 10.1038/s41598-019-43803-5

**Published:** 2019-05-17

**Authors:** A. Azcorra, L. F. Chiroque, R. Cuevas, A. Fernández Anta, H. Laniado, R. E. Lillo, J. Romo, C. Sguera

**Affiliations:** 1 Universidad Carlos III de Madrid, Leganés, Madrid, Spain; 2 IMDEA Networks Institute, Leganés, Madrid, Spain; 3 Department of Mathematical Sciences, Universidad EAFIT, Medellín, Colombia; 4 Department of Statistics, Universidad Carlos III de Madrid, Madrid, Spain; 5 UC3M-BS Institute of Financial Big Data, Universidad Carlos III de Madrid, Madrid, Spain

Correction to: *Scientific Reports* 10.1038/s41598-018-24874-2, published online 03 May 2018

The original version of this Article contained an error in Affiliation 3, which was incorrectly given as ‘Department of Mathematical Sciences, Universidad EAFIT, Universidad Nacional de Colombia, Medellín, Colombia’. The correct affiliation is listed below:

Department of Mathematical Sciences, Universidad EAFIT, Medellín, Colombia

This error has now been corrected in the HTML and PDF versions of the Article and in the accompanying Supplementary Material file.

