# Supplementary material for: Unsupervised Scalable Statistical Method for Identifying Influential Users in Online Social Networks
Source: Sci Rep. 2018 May 3;8:6955. doi: 10.1038/s41598-018-24874-2 (PMC5934471; doi:10.1038/s41598-018-24874-2)
Supplement: Supplementary file 1 — Supplementary Material [file 41598_2018_24874_MOESM1_ESM.pdf]

Unsupervised Scalable Statistical Method for Identifying Influential Users in Online Social Networks
Supplementary Material

Azcorra, A.[1,2], Chiroque, L. F.[1,2], Cuevas, R.[1], Fernández Anta, A.[2], Laniado, H.[3], Lillo, R. E.[4,5], Romo, J.[4,5], and Sguera, C.[5]

[1]Universidad Carlos III de Madrid, Leganés, Madrid, Spain
[2]IMDEA Networks Institute, Leganés, Madrid, Spain
[3]Department of Mathematical Sciences, Universidad EAFIT, Medellín, Colombia
[4]Department of Statistics, Universidad Carlos III de Madrid, Madrid, Spain
[5]UC3M-BS Institute of Financial Big Data, Universidad Carlos III de Madrid, Madrid, Spain

1 Introduction

In this work we deal with real multivariate data that describes $d = 21$ features of Google+ users. See Section 6 for a detailed description of the features. Moreover, note that, after a preliminary analysis of the data, we run our study using only 21 features.

When multivariate data have more than three dimensions is practically impossible to graphically visualize the observations using Cartesian orthogonal axes. A convenient alternative is given by a visualization tool known as parallel coordinates ([30]). Parallel coordinates substitute Cartesian orthogonal axes with parallel axes, represent a multivariate point on these parallel coordinates by a series of points and then connect each pair of adjacent points by a line, forming at the end a broken line.

As suggested by [19], once represented by means of parallel coordinates, observations $\mathbf{x} \in \mathbb{R}^d$ can be seen as real functions defined on an arbitrary set of equally spaced domain points, e.g., $\{1, \ldots, d\}$, and $\mathbf{x}$ can be expressed as $x = \{x(1), \ldots, x(d)\}$. Note that observations that can be represented as curves then can be analyzed using the tools provided by an area of statistics known as functional data analysis (FDA*). We use the same approach in this work: first, we represent multivariate data using parallel coordinates; second, we treat the transformed multivariate data as functional. Note that this approach provides a unified framework where both visualization and analysis tools become available.

In the univariate or multivariate framework we assume that observations are generated by an univariate or multivariate random variable, accordingly. Similarly, in the FDA framework it is common to assume that observations are generated by a functional random variable $X \in \mathbb{F}$, where $\mathbb{F}$ is a functional space. An alternative way to interpret X is as a stochastic process $\{X(t), t \in I\}$, where I is an interval in $\mathbb{R}$.

The accurate identification of outliers is an important aspect in any statistical data analysis and scientists have interest in identifying such atypical observations because:

- a statistical analysis performed on a sample containing outliers may lead to misleading results;
- a non-statistical analysis of the observations detected as outliers done by experts in the field may reveal interesting aspects of the phenomenon under study.

*For overviews on FDA, see [31], [32], [33] or [34]

Outlier detection is an issue also in FDA, where an outlier can be defined as an observation generated by a functional random variable with a different distribution than the one generating the observations of a functional sample ([13]). Note that the previous definition covers the case of isolated outliers, which exhibit outlying behavior during a very short domain interval, and the case of persistent outliers, which are outlying on a large part of the domain ([12]). In this work we focus on persistent outliers, and on the three types of persistent outliers defined by [12]: *shift/magnitude* outliers, which have the same shape of the majority, but are moved away; *amplitude* outliers, curves that may have the same shape as the majority but their scale differs; *shape* outliers, curves whose shape differs from the majority.

We propose a statistical methodology for identifying outliers and classifying them as magnitude, amplitude and shape outliers, and we argue that influential users of Online Social Networks can be seen as outliers since they usually show behaviors and patterns that are different from those of non-influential users. Therefore, the new methodology can be used to search for potentially relevant Google+ users, whose identification will be based on a statistical criterion instead of directed arguments [10, 8, 11, 9, 4].

Detection of influencers may have a special relevance in digital marketing in order to maximize profits using social networks data [35, 36]. Moreover, since the new procedure searches for three different types of outliers, it may reveal different types of relevant Google+ users. We refer to the proposed method as Massive Unsupervised Outlier Detection (MUOD).

Before formally defining MUOD, let us consider the illustrative example in Figure S1, where observations are curves having the following characteristic: at different but relatively common levels, they all share the same behavior in terms of magnitude, amplitude and shape. More precisely, the curves in Figure S1 represent the same equation, but at different levels defined by different constants, and we can state that data set in Figure S1 does not contain any outlier.

Figure S2 is a modification of Figure S1, where, using now relatively large constants, we obtain several curves that can be seen as magnitude outliers, without being amplitude and shape outliers. One of the goal of MUOD is to detect this kind of outliers and identify them as magnitude outliers.

Figures S3 and S4 are two additional modifications of Figure S1, and they serve to

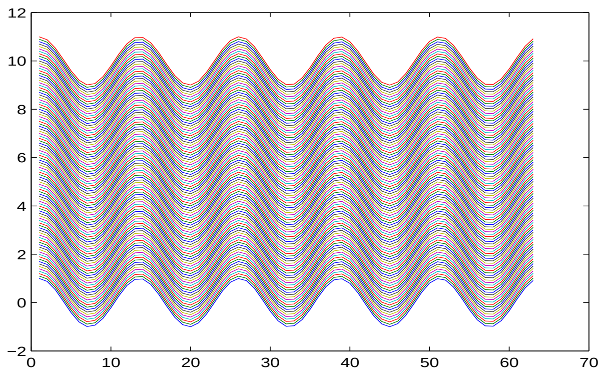

Figure S1: Illustrative Example 1.

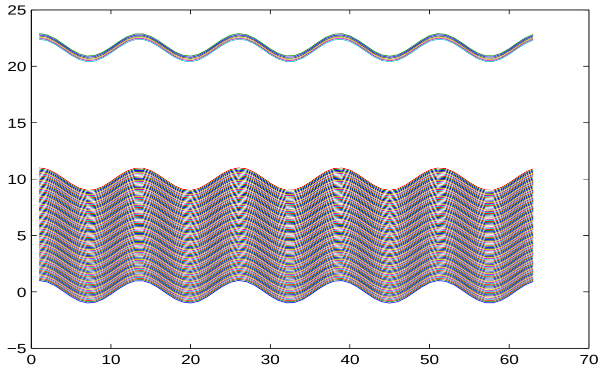

Figure S2: A modification of Example 1 allowing magnitude outliers.

illustrate examples of amplitude and shape contamination of a data set. Our proposal will also deal with these types of scenarios of contamination, trying to solve their associated outlier detection problems.

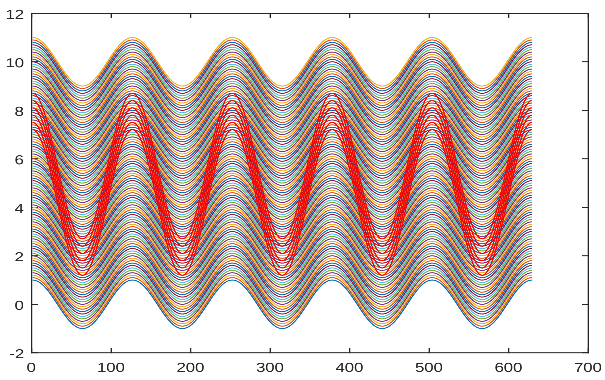

Figure S3: A modification of Example 1 allowing amplitude outliers.

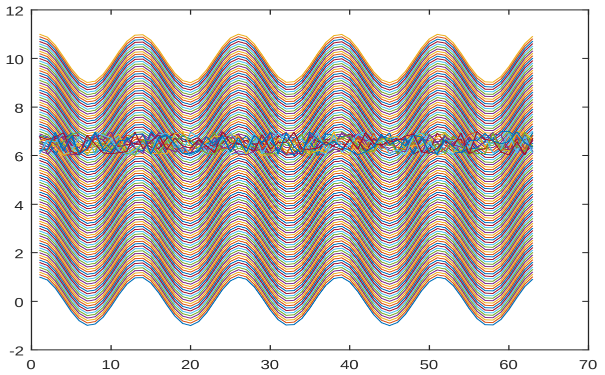

Figure S4: A modification of Example 1 allowing shape outliers.

Finally, note that we also aim to satisfactorily solve outlier detection problems where magnitude, amplitude and shape outliers simultaneously contaminate a data set as in Figure S5, or even where an outlier can belong to several classes.

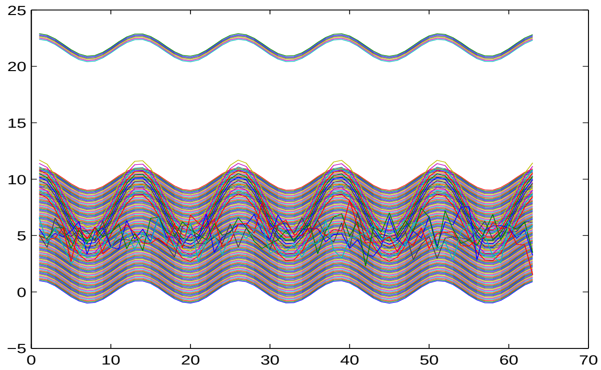

Figure S5: A modification of Example 1 allowing magnitude, amplitude and shape outliers.

2 A New Outlier Detection Method Based on Indices of Magnitude, Amplitude and Shape

In this section we present three indices that can be used to analyze the type of data sets that we are considering in this work, i.e., multivariate data sets represented by means of a parallel coordinates system. These indices can be interpreted as similarity measures of an observation with respect to a sample, and each one of them focuses on a different feature of the data: magnitude, amplitude or shape. We start introducing the shape index, which is based on a well known statistical coefficient such as the Pearson correlation coefficient.

Let $\mathcal{X} = \{x_1, x_2, \ldots, x_n\}$ be a set of n curves whose common discretized form is defined on a given set of d equally-spaced domain points, and let x be another curve defined on the same set. We define the shape index of x with respect to $\mathcal{X}$ as

$$I_S(x, \mathcal{X}) = \left| \frac{1}{n} \sum_{j=1}^{n} \rho(x, x_j) - 1 \right|, \quad (1)$$

where $\rho(x, x_j)$ is the Pearson correlation coefficient between of x and x_j, and it is the measure responsible to capture the similarity between x and x_j in terms of shape. Note that, if we compute (1) for each curve in Figure S1 with respect to the whole data set, then we always obtain an I_S equal to 0. The same happens with the data sets in Figures S2 and S3. However, if we compute (1) for one of the shape outliers from Figure S4, say x_s, with respect to the whole data set, then we obtain $I_S(x_s, \mathcal{X})$ significantly greater than 0, while $I_S(x_c, \mathcal{X}) \simeq 0$,

where x_c is any of the "common" curves in Figure S4. Indeed, we have computed $I_S(x, \mathcal{X})$ for all the curves in Figure S4 and sorted them from the lowest to the highest indices, that is, we have ranked the curves using $I_S(x, \mathcal{X})$ as criterion. Figure S6 is a scatter plot of the $I_S(x, \mathcal{X})$-based ranks (horizontal axis) and the $I_S(x, \mathcal{X})$ values (vertical axis): there are 12 points that stand out because of their $I_S(x, \mathcal{X})$ value, and they correspond exactly to the 12 shape outliers that we have generated in Figure S4.

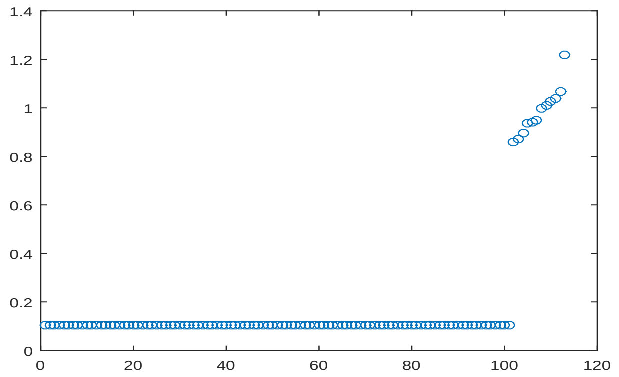

Figure S6: Scatter plot of the $I_S(x, \mathcal{X})$-based ranks (horizontal axis) and the $I_S(x, \mathcal{X})$ values (vertical axis) for the curves from Figure S4.

Therefore, I_S seems to help in detecting shape outliers. Clearly, in real outlier detection problems the behavior of both non-outlying and outlying curves is less "regular" than the one in our illustrative examples. Hence, the corresponding scatter plots will show more differences among the index values of non-outlying curves, and likely less differences between the index values of non-outlying and outlying curves. However, we hold our statement: I_S is a useful tool to identify shape outliers.

The magnitude and amplitude indices are also based on some well-known concepts in statistics. Let $\hat{\alpha}_j$ and $\hat{\beta}_j$ be the estimated intercept and slope of a simple linear regression model where the discretized version of x represents the observed values of the dependent variable and the discretized version of x_j represents the observed values of the regressor. More formally,

$$\hat{\beta}_j = \frac{Cov(x, x_j)}{Var(x_j)}, \qquad \hat{\alpha}_j = \overline{x} - \hat{\beta}_j \overline{x}_j. \tag{2}$$

Then, we define the magnitude index of x with respect to $\mathcal{X}$ as

$$I_M(x, \mathcal{X}) = \left| \frac{1}{n} \sum_{j=1}^{n} \hat{\alpha}_j \right|, \tag{3}$$

and the amplitude index of x with respect to $\mathcal{X}$ as

$$I_A(x, \mathcal{X}) = \left| \frac{1}{n} \sum_{j=1}^{n} \hat{\beta}_j - 1 \right|. \tag{4}$$

As for I_S, using I_M we compute $I_M(x, \mathcal{X})$ for all the curves in Figure S2 and obtain the scatter plot of Figure S7. Also in this case we observe points that stand out because of their index's values, which correspond to the magnitude outliers from Figure S2.

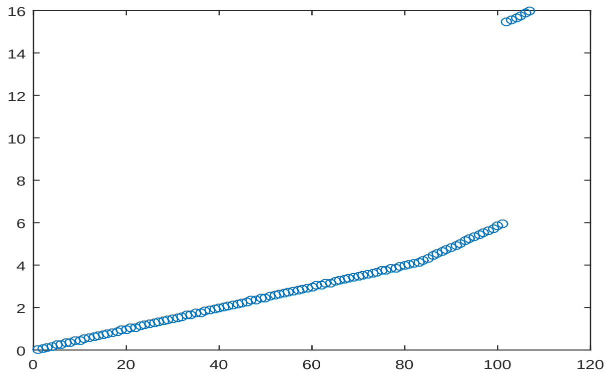

Figure S7: Scatter plot of the $I_M(x, \mathcal{X})$-based ranks (horizontal axis) and the $I_M(x, \mathcal{X})$ values (vertical axis) for the curves from Figure S2.

Similarly, using I_A we compute $I_A(x, \mathcal{X})$ for all the curves in Figure S3 and obtain the scatter plot shown in Figure S8. Once more, the true outliers, i.e., the amplitude outliers from Figure S3, stand out in the scatter plot.

Therefore, also I_M and I_A seem to help in detecting the type of outliers for which they were designed, i.e., magnitude and amplitude outliers, respectively.

Now, we have to be able to detect and filter those values which stand out in the scatter plots. To do so, we can use a heuristic such as the first derivative of the indices curve (if necessary, normalized) and filtering out those fulfilling a certain condition (e.g. slope $\geq$ 2). Also, we can apply the *tangent method* as described below. Let $I_S(\mathcal{X}) = \{I_S(x_1, \mathcal{X}), \cdots, I_S(x_n, \mathcal{X})\}$ be the set of shape indices. Analogously, let $I_M(\mathcal{X})$ and $I_A(\mathcal{X})$ be the set of magnitude and amplitude indices respectively. Hereafter, we will use $I(\mathcal{X})$

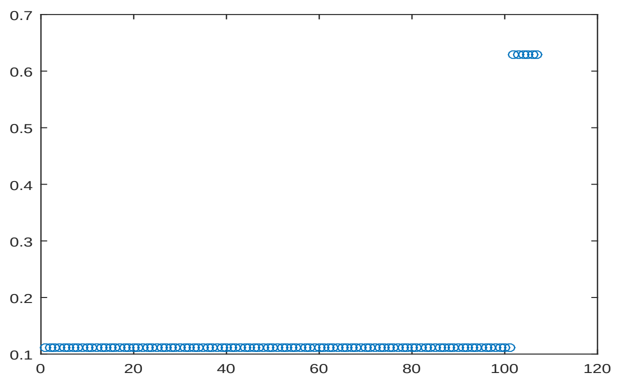

Figure S8: Scatter plot of the $I_A(x, \mathcal{X})$-based ranks (horizontal axis) and the $I_A(x, \mathcal{X})$ values (vertical axis) for the curves from Figure S3.

for any of these three sets of indices indistinctly. Then, we sort (ascending order) and plot the set $I(\mathcal{X})$ as in, e.g, Figures S6 to S8. Thus, the *tangent method* consists in taking, as boundary between regular curves and outliers, the value $I^*(\mathcal{X}) = I(x^*, \mathcal{X})$ at the intersection point x^* between the tangent at the furthest right point and the horizontal axis. Figure S9 illustrate this method. The tangent method is inspired on the classical scale determination assuming you have an exponential decay, as described in [14]. Besides, the authors compare this method with the first derivative one, being the latter outperformed by the tangent method.

Finally, we use the value $I^*(\mathcal{X})$ to identify the outstanding indices. The threshold $I^*(\mathcal{X})$ represents the maximum index value to be considered a non-outlying value. Hence, any index value above this threshold is considered an outlier. Thus, we obtain the set of outliers as $I_{out}(\mathcal{X}) = \{I(x_i, \mathcal{X}) : I(x_i, \mathcal{X}) > I^*(\mathcal{X})\}$.

3 A New Outlier Detection Method: Implementation

We have built a scalable algorithm that implements the method. Now, we are going to outline the implementation details of our algorithm and how it scales. Since we have the set $\mathcal{X} = \{x_1, x_2, \ldots, x_n\}$, where $|x_i| = d$, we build the row-matrix $\mathbf{M}_{\mathcal{X}} = [x_1\ x_2 \ldots x_{n-1}\ x_n] \in \mathbb{R}^{d \times n}$, where $x_i \in \mathbb{R}^d$ is a curve representing each user in our dataset. For scalability, we assume $d \ll n$.

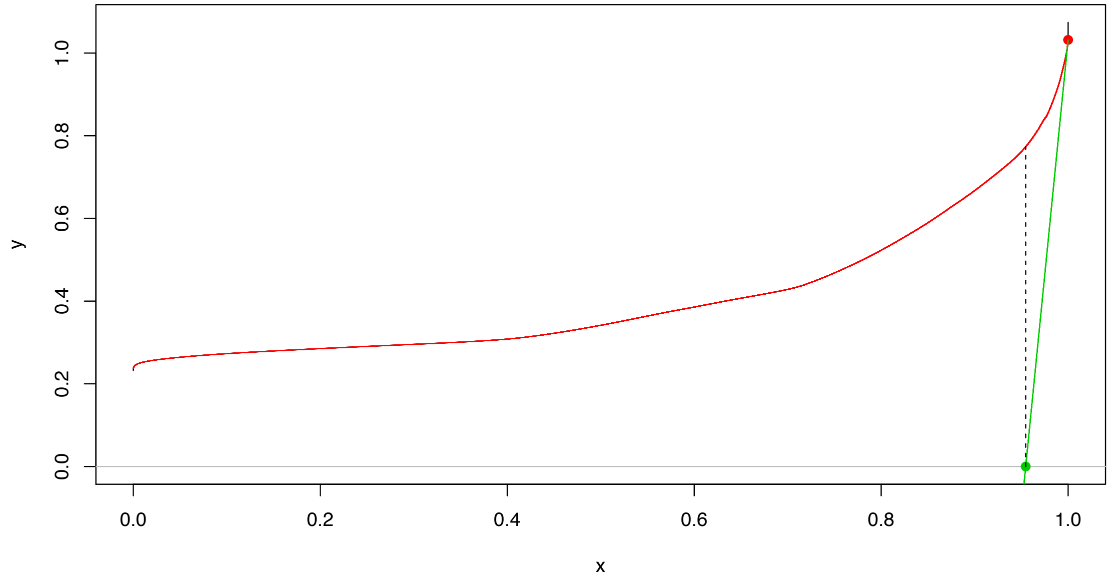

Figure S9: Tangent method applied to shape indices for our real dataset. The horizontal axis represents sample percentiles based on the shape index. The vertical axis represents shape index values. The green dot cuts the x-axis at $x^* = 0.9546$, yielding a 4.538% of outliers (the higher values at the right-side). $I^*(\mathcal{X}) = I(0.9546, \mathcal{X})$.

Partitioning A key step to make our algorithm *parallel* and, consequently, *scalable* is to partition the matrix $\mathbf{M}_{\mathcal{X}}$. This way, we can simultaneously compute the indices leveraging the current multi-core machines. For this purpose, we define a partition $\Pi = \{\pi_j\}$ of users, where $\pi_j \subseteq \mathcal{X}$ are non empty, mutually disjoint and $\bigcup_j \pi_j = \mathcal{X}$. The number of classes is $k = |\Pi|$. That will allow us to separately compute the indices in each class. Note that different partitions may exist and they range from the discrete partition ($k = n : |\pi_j| = 1, \forall j$) to the unit partition ($k = 1 : |\pi_j| = n, j = \{1\}$). The partitions considered hereinafter are equal sized and always consecutive elements (users), according to the matrix $\mathbf{M}_{\mathcal{X}}$. Our algorithm has the matrix $\mathbf{M}_{\mathcal{X}}$ and the correspondant set of users π_j as input. Note that, we can see $\pi_j \in \Pi$ as a matrix $\pi_j = [x_i \dots x_{i+|\pi_j|}] \in \mathbb{R}^{d\times|\pi_j|}$ for every j according to the partition Π.

We describe now the different sub-algorithms for the computation of the indices. Note that the algorithms will use any set π_j as input, regardless of the partition chosen.

3.1 *Shape* Indices

We present the algorithm to compute $I_S(\pi_j, \mathcal{X})$. Algorithm 1 shows a possible implementation of Equation 1. The *mean* function in Line 3 is a column-wise mean on the matrix *cor*.

Algorithm 1 Get Shape Indices

1: **function** GET_SHAPE_INDICES($\mathbf{M}_{\mathcal{X}}$, π_j)
2: $\quad$ **cor** $\leftarrow \rho(\mathbf{M}_{\mathcal{X}}, \pi_j) : \mathbb{R}^{d\times n} \times \mathbb{R}^{d\times|\pi_j|} \rightarrow \mathbb{R}^{n\times|\pi_j|}$
3: $\quad$ Return $|mean(\mathbf{cor}) - \mathbf{1}| \equiv I_S(\pi_j, \mathcal{X}) : \mathbb{R}^{n\times|\pi_j|} \rightarrow \mathbb{R}^{|\pi_j|}$

Time Complexity The algorithm's complexity is dominated by computing the correlation (ρ) matrix **cor** whose complexity is $\mathcal{O}(|\pi_j|dn)$. Since this function will be run for every set π_j it will be computed for the n curves/users at the end. However, it may be computed in parallel in up to p cores, which yields a final time complexity of $\mathcal{O}(\frac{n^2d}{p})$

3.2 *Magnitude* & *Amplitude* Indices

These two indices are jointly computed since they are estimated together from a linear regression model as explained in Section 2 (see equation (2)).

Algorithm 2 shows a possible implementation for computing $I_M(\pi_j, \mathcal{X})$ and $I_A(\pi_j, \mathcal{X})$. The $mean(\cdot)$ functions in lines 3, 8, 10 and 12 are column-wise. Finally, the division and product operations in lines 7 and 9 are both column and element-wise, while the operation in line 11 is row-wise.

Algorithm 2 Get Magnitude&Amplitude Indices

1: **function** GET_MAGAMP_INDICES($\mathbf{M}_{\mathcal{X}}$, π_j, *means*, *vars*)
2: *Paramaters*:
3: $\quad means \leftarrow mean(\mathbf{M}_{\mathcal{X}}) : \mathbb{R}^{d \times n} \rightarrow \mathbb{R}^n$
4: $\quad vars \leftarrow Var(\mathbf{M}_{\mathcal{X}}) \equiv \{Var(x_1), \ldots, Var(x_n)\} : \mathbb{R}^{d \times n} \rightarrow \mathbb{R}^n$
5: *algorithm*:
6: $\quad \mathbf{cov} \leftarrow Cov(\mathbf{M}_{\mathcal{X}}, \pi_j) : \mathbb{R}^{d \times n} \times \mathbb{R}^{d \times |\pi_j|} \rightarrow \mathbb{R}^{n \times |\pi_j|}$
7: $\quad \boldsymbol{\beta} \leftarrow \mathbf{cov}/vars : \mathbb{R}^{n \times |\pi_j|} \times \mathbb{R}^n \rightarrow \mathbb{R}^{n \times |\pi_j|}$
8: $\quad amp \leftarrow |mean(\boldsymbol{\beta}) - \mathbf{1}| \equiv I_A(\pi_j, \mathcal{X}) : \mathbb{R}^{n \times |\pi_j|} \rightarrow \mathbb{R}^{|\pi_j|}$
9: $\quad \boldsymbol{\beta}\mathbf{x} \leftarrow \boldsymbol{\beta} \cdot means : \mathbb{R}^{n \times |\pi_j|} \times \mathbb{R}^n \rightarrow \mathbb{R}^{n \times |\pi_j|}$
10: $\quad \overline{\pi_j} \leftarrow mean(\pi_j) : \mathbb{R}^{d \times |\pi_j|} \rightarrow \mathbb{R}^{|\pi_j|}$
11: $\quad \boldsymbol{\alpha} \leftarrow -\boldsymbol{\beta}\mathbf{x} + \overline{\pi_j} : \mathbb{R}^{n \times |\pi_j|} \times \mathbb{R}^{|\pi_j|} \rightarrow \mathbb{R}^{n \times |\pi_j|}$
12: $\quad mag \leftarrow |mean(\boldsymbol{\alpha})| \equiv I_M(\pi_j, \mathcal{X}) : \mathbb{R}^{n \times |\pi_j|} \rightarrow \mathbb{R}^{|\pi_j|}$
13: $\quad$ Return $[mag\ amp] \equiv [I_M(\pi_j, \mathcal{X})\ I_A(\pi_j, \mathcal{X})]$

Time Complexity The algorithm's complexity is dominated by computing the covariance matrix **cov** whose complexity is $\mathcal{O}(|\pi_j|dn)$. Similarly as in the previous algorithm, this function will be run for every set π_j and hence, it will be computed for the n curves/users at the end. However, it may be computed in parallel in up to p cores, which again yields a final time complexity of $\mathcal{O}(\frac{n^2 d}{p})$

3.3 Three indices simultaneously

We have already shown how to compute the three indices with a total complexity of $\mathcal{O}(\frac{n^2 d}{p})$ in the two cases, which would give us the same total time complexity. However, we would like to compute the three indices simultaneously in order to optimize the time spent. For this purpose, we are going to leverage the following result.

$$\rho(\mathbf{x},\mathbf{y}) = \frac{Cov(\mathbf{x},\mathbf{y})}{\sigma_x \sigma_y} \tag{5}$$

Thus, we can compute the correlation matrix used in Algorithm 1 from the covariance matrix used in Algorithm 2. Basically, the resulting algorithm is similar to the previous one for magnitude and amplitude indices, but with one more parameter and one more line. This extra Line 8 applies the Equation 5, first dividing (column-wise) by the standard deviations of $\mathbf{M}_{\mathcal{X}}$, and second dividing (row-wise) by the standard deviations from the corresponding curves in π_j. Algorithm 3 below outlines our proposed method.

Algorithm 3 Get Shape, Magnitude and Amplitude Indices

1: **function** GET_SMA_INDICES($\mathbf{M}_{\mathcal{X}}$, π_j, *means*, *vars*, *sds*)
2: *Paramaters*:
3: $\quad$ $means \leftarrow mean(\mathbf{M}_{\mathcal{X}}) : \mathbb{R}^{d\times n} \to \mathbb{R}^n$
4: $\quad$ $vars \leftarrow Var(\mathbf{M}_{\mathcal{X}}) \equiv \{Var(x_1),\ldots,Var(x_n)\} : \mathbb{R}^{d\times n} \to \mathbb{R}^n$
5: $\quad$ $sds \leftarrow \sigma(\mathbf{M}_{\mathcal{X}}) \equiv \{\sigma(x_1),\ldots,\sigma(x_n)\} : \mathbb{R}^{d\times n} \to \mathbb{R}^n$
6: *algorithm*:
7: $\quad$ $\mathbf{cov} \leftarrow Cov(\mathbf{M}_{\mathcal{X}}, \pi_j) : \mathbb{R}^{d\times n} \times \mathbb{R}^{d\times|\pi_j|} \to \mathbb{R}^{n\times|\pi_j|}$
8: $\quad$ $\mathbf{cor} \leftarrow \mathbf{cov}/sds/sds[j] : \mathbb{R}^{n\times|\pi_j|} \times \mathbb{R}^n \times \mathbb{R}^{|\pi_j|} \to \mathbb{R}^{n\times|\pi_j|}$
9: $\quad$ $sha \leftarrow |mean(\mathbf{cor}) - \mathbf{1}| \equiv I_S(\pi_j,\mathcal{X}) : \mathbb{R}^{n\times|\pi_j|} \to \mathbb{R}^{|\pi_j|}$
10: $\quad$ $\boldsymbol{\beta} \leftarrow \mathbf{cov}/vars : \mathbb{R}^{n\times|\pi_j|} \times \mathbb{R}^n \to \mathbb{R}^{n\times|\pi_j|}$
11: $\quad$ $amp \leftarrow |mean(\boldsymbol{\beta}) - \mathbf{1}| \equiv I_A(\pi_j,\mathcal{X}) : \mathbb{R}^{n\times|\pi_j|} \to \mathbb{R}^{|\pi_j|}$
12: $\quad$ $\boldsymbol{\beta}\mathbf{x} \leftarrow \boldsymbol{\beta} \cdot means : \mathbb{R}^{n\times|\pi_j|} \times \mathbb{R}^n \to \mathbb{R}^{n\times|\pi_j|}$
13: $\quad$ $\overline{\pi_j} \leftarrow mean(\pi_j) : \mathbb{R}^{d\times|\pi_j|} \to \mathbb{R}^{|\pi_j|}$
14: $\quad$ $\boldsymbol{\alpha} \leftarrow -\boldsymbol{\beta}\mathbf{x} + \overline{\pi_j} : \mathbb{R}^{n\times|\pi_j|} \times \mathbb{R}^{|\pi_j|} \to \mathbb{R}^{n\times|\pi_j|}$
15: $\quad$ $mag \leftarrow |mean(\boldsymbol{\alpha})| \equiv I_M(\pi_j,\mathcal{X}) : \mathbb{R}^{n\times|\pi_j|} \to \mathbb{R}^{|\pi_j|}$
16: $\quad$ Return $[sha\ mag\ amp] \equiv [I_S(\pi_j,\mathcal{X})\ I_M(\pi_j,\mathcal{X})\ I_A(\pi_j,\mathcal{X})]$

Time Complexity This final algorithm's complexity does not increase from the previous one, $\mathcal{O}(\frac{n^2 d}{p})$. In spite of not decreasing the time complexity, this last algorithm computes one more index adding a few more instructions, saving the whole computation of the correlation matrix (from scratch) in Algorithm 1. Additionally, each algorithm may be computed in parallel for each partition π_j and then concatenating the results. Observe that, we can reduce the time complexity depending on how many partitions we use and how much computing power we had, i.e., $p = 10$ leads to one order of magnitude reduction, while $p = 100$ allows two orders of magnitude reduction, and so on.

4 Outlier Detection in Functional Data

The aim of this section is to present the results of a simulation study where we compare MUOD with some competitors recently used in the literature of functional data. Since some of them are based on the use of measures known as functional depths, we briefly introduce a general definition. A functional depth is a measure that allows to order and rank the observations in a functional sample from the most to the least central, and it gives high values to central observations and low values to non-central observations. In FDA, unlike in univariate statistics, where $\mathbb{R}$ provides a natural order criterion for observations, several criteria have been employed to order functional data, and therefore there exist different implementations of the notion of functional depth (see [37], where different functional depths have been reviewed and used in classification problems).

Recall that we propose three type-oriented outlier detection methods to find alternatively magnitude (MUOD_{mag}), amplitude (MUOD_{amp}) and shape (MUOD_{sha}) outliers, and note that we refer to their simultaneous use on a single data set simply as MUOD. We will compare MUOD_{mag}, MUOD_{amp}, MUOD_{sha} and MUOD with a battery of competitors given by the outlier detection methods considered in [38]. In addition, we also consider a method proposed in [39]. Next, we briefly describe these competitors (for more details, see [38] and references therein, and [39]):

- B_{tri} and B_{wei} ([13]): depth-based outlier detection methods which select the threshold of a functional depth by means of two alternative procedures based on bootstrap techniques. B_{tri} and B_{wei} differ in their resampling steps: in B_{tri} the resampling is done on a trimmed version of the original sample, that is, after deleting from the sample a given proportion of least deep curves (trimmed resampling); in B_{wei} the resampling is done giving weights to sample observations that are proportional to their depth values (weighted resampling). B_{tri} and B_{wei} work with any functional depth. Following their authors, we use B_{tri} and B_{wei} together with the h-modal depth ([40]).

- FBAG ([41]): procedure that reduces the outlier detection problem from functional to multivariate by means of the functional principal component analysis technique. Once obtained the first two functional principal components scores, FBAG orders the scores

using the multivariate halfspace depth ([42]) and builds a non-outlying region. FBAG detects as outliers those observations whose scores are outside the non-outlying region.

- FHDR ([41]): procedure that differs from FBAG after obtaining the first two functional principal components scores. FHDR performs a bivariate kernel density estimation on the scores and defines a high density region. FHDR detects as outliers those observations whose scores are outside the high density region.

- FBPLOT ([18]): procedure that extends the standard boxplot to the functional framework, allowing the identification of a non-outlying band defined by a specified percentage of the deepest curves, and therefore the detection of outliers. FBPLOT works with any functional depth. Following its authors, we use FBPLOT together with the modified band depth ([19]).

- OG ([39]): depth-based outlier detection method based on a visualization tool known as outliergram. The outliergram exploits the relation between the modified band depth ([19]) and modified epigraph index ([43]) to help understanding shape features of observations, and it expressly detects shape outliers.

- KFSD_{smo}, KFSD_{tri} and KFSD_{wei} ([38]): depth-based outlier detection methods which select the threshold of the kernelized functional spatial depth (KFSD, [37]) by means of a probabilistic procedure based on three alternative resampling techniques. KFSD_{smo}, KFSD_{tri} and KFSD_{wei} differ in their resampling steps: in KFSD_{smo} the resampling is simple and smoothed, that is, once an observation is sampled, a small perturbation is added to the observation to avoid the presence of repeated observations; in KFSD_{tri} the resampling is trimmed and smoothed; in KFSD_{wei} the resampling is weighted and smoothed.

We have tested all these methods with random samples of our dataset of Google+ users in order to observe the time performance. Experiments have been carried out in an AMD Opteron 6276 multicore (x64) @ 2.3GHz with 512GiB of RAM under Debian 7.9. Figure S10 shows the time performance for all these methods including ours. We have run our method with one single partition (MUOD), and using 10 partitions (MUOD 10-way) in order to check the scalability and verify that the running time decreases one order of magnitude (better

time performance). It is observable that the parallel version needs a setup time which is amortized as long as the data size increases.

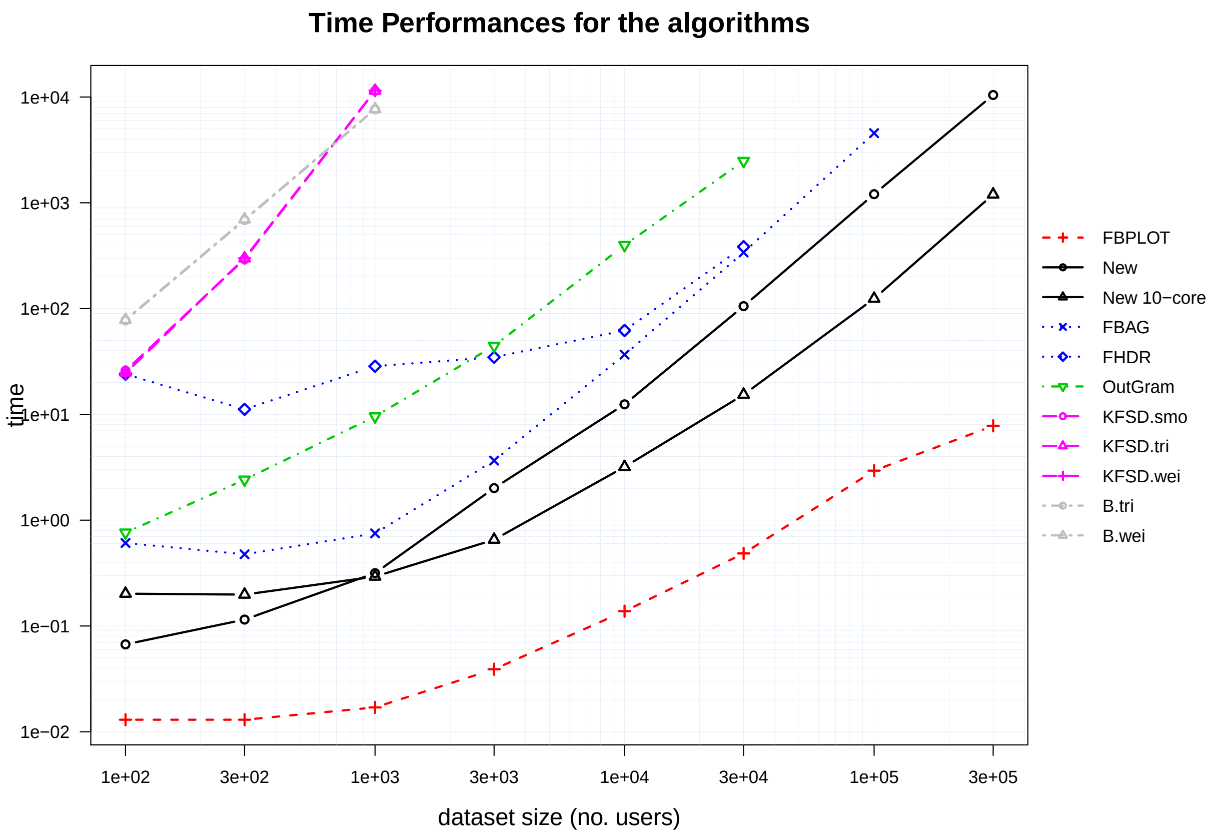

Figure S10: Time performance for the different algorithms computing different size of data (log-log scale). FBPLOThas the best performance by far as we increase the data size. MUOD and MUOD 10-way are the second best performance lines. Observe that, FBPLOTand MUOD are the only ones performing in less than 100 seconds for a data size of $3 \cdot 10^4$ users. FBAGand FHDRhave a similiar behaviour as the data size grows and performs similar to MUOD, but they are not able to process a data size of $3 \cdot 10^5$. OutGRAM have a similar behaviour than the former algorithms but is not able to process dataset bigger than $3 \cdot 10^4$ users. Then we found the KSFD family and the B family with a poor performance and a dataset size limit of 10^3 users.

We conclude that, only FBPLOT and MUOD are able to scale out for datasets bigger than 10^5 users, which is far from our real dataset from Google+ (5, 619, 786 users). For this reason, we are only going to use FBPLOT and MUOD in our real data study case (Section 6), where both algorithms are evaluated and compared.

5 Simulation Study

In this section we present the results of a simulation study where we compare our methods with the competitors described in the previous section. The goal is to show that our procedures are competitive in the usual framework used in the FDA literature.

5.1 Mixture Models 1, 2, and 3

In the first part of our simulation study we generate observations using the following model:

$$X(t) = a_1 \sin t + a_2 \cos t, \tag{6}$$

where $t \in \{t_j\}$, $\{t_j\}$ is a sequence of d equidistant points between 0 and 2π, and a_1 and a_2 are independent realizations of a continuous uniform random variable between 0.75 and 1.25. In Figure S11 we show a simulated data set obtained using (6), $d = 25$ and $n = 100$, where n is the size of the data set.

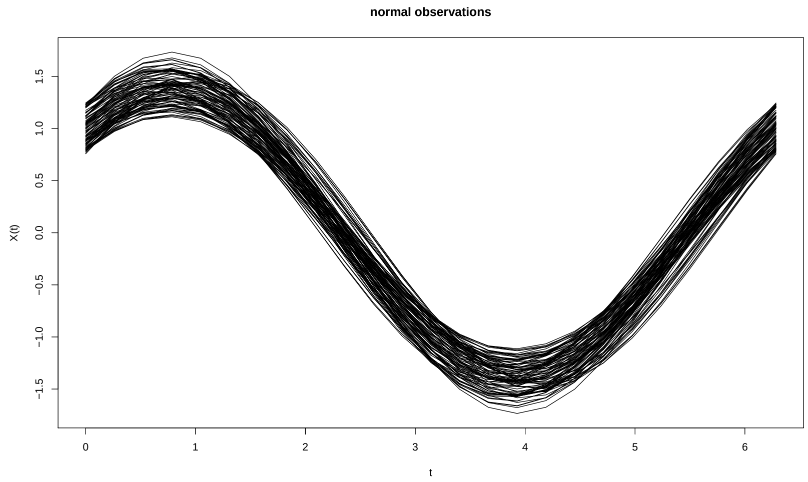

Figure S11: Simulated observations.

To generate outliers with respect to model (6) we use three different models. They generate magnitude, amplitude, and shape outliers, respectively. The magnitude outliers' model is given by

$$X(t) = a_1 \sin t + a_2 \cos t + k0.5, \tag{7}$$

where k is a realization of a discrete random variable with $P(k = -1) = P(k = 1) = 0.5$. In Figure S12 we show a simulated data set obtained using a mixture of models (6) and (7) (mixture model 1), with $\alpha = 0.05$ being the probability that each curve is a magnitude outlier.

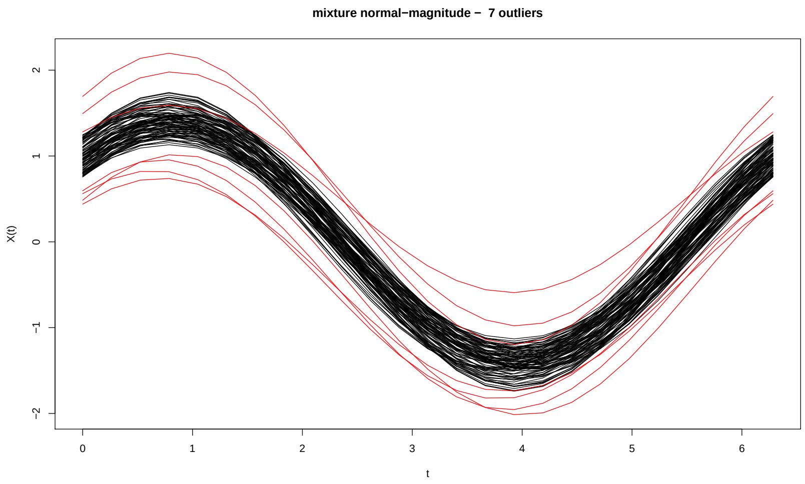

Figure S12: Mixture model 1: magnitude outliers.

The amplitude outliers' model is given by

$$X(t) = b_1 \sin t + b_2 \cos t, \tag{8}$$

where b_1 and b_2 are independent realizations of a continuous uniform random variable between 1.30 and 1.50. In Figure S13 we show a simulated data set obtained using a mixture of models (6) and (8) (mixture model 2), with $\alpha = 0.05$ being the probability that each curve is an amplitude outlier.

The shape outliers' model is given by

$$X(t) = a_1 \sin t + a_2 \cos t + \epsilon(t), \tag{9}$$

where $\epsilon(t) \in \{\epsilon(t_j)\}$ and $\{\epsilon(t_j)\}$ is a d-dimensional sample of independent realizations of a normal random variable having mean equal to 0 and variance equal to $1/9$. In Figure S14 we show a simulated data set obtained using a mixture of models (6) and (9) (mixture model 3), with $\alpha = 0.05$ being the probability that each curve is a shape outlier.

For each mixture model, the comparison among methods is based on 100 generated data

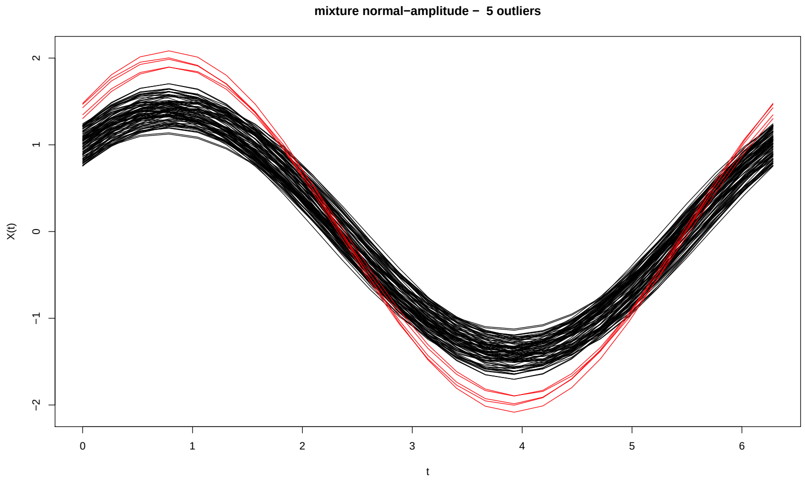

Figure S13: Mixture model 2: amplitude outliers.

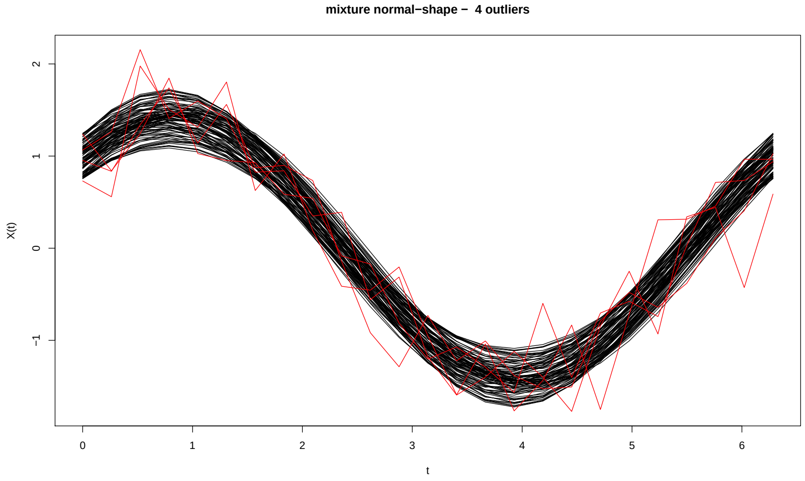

Figure S14: Mixture model 3: shape outliers.

sets and is performed in terms of correct outlier detection percentages, false outlier detection percentages and F-measures. F-measures are given by

$$F = \frac{2RP}{R + P}, \tag{10}$$

where $R = \frac{TP}{(TP+FN)}$ is known as recall measure, $P = \frac{TP}{TP+FP}$ is known as precision measure and TP, FN, and FP are the number of true positives, false negatives and false positives, respectively.

The F-measure is the harmonic mean of precision and recall and it is widely used instead of accuracy when the data set is unbalanced as in our seting ([44]). It gives a good trade-off for precision while preserving a high recall rate.

The results obtained by our methods and their competitors are shown in Table S1, where we also report the F-measure-based rankings of the methods for each mixture model.

Table S1: Correct outlier detection percentages (c), false outlier detection percentages (f), F-measures (F) and F-measure-based rankings of the methods (r) in mixture models 1, 2 and 3 which allow for magnitude (*mag*), amplitude (*amp*) and shape (*sha*) outliers, respectively.

	mag				*amp*				*sha*			
	c	f	F	r	c	f	F	r	c	f	F	r
B_{tri}	54.55	0.00	0.71	6	16.67	0.01	0.29	9	83.82	0.00	0.91	2
B_{wei}	98.42	0.05	0.98	1	25.00	0.01	0.40	8	100.00	0.00	1.00	1
FBAG	3.16	0.27	0.06	10	91.67	0.46	0.91	2	8.29	0.24	0.14	11
FHDR	15.61	4.43	0.16	9	75.97	1.14	0.77	6	24.08	3.96	0.24	10
FBPLOT	39.13	0.00	0.56	8	0.39	0.00	0.00	11	64.55	0.00	0.79	9
OG	0.00	0.00	-	-	0.78	0.00	0.02	10	0.00	0.00	-	-
$KFSD_{smo}$	98.81	0.09	0.98	1	82.17	0.11	0.89	3	84.39	0.13	0.90	3
$KFSD_{tri}$	99.60	2.51	0.81	4	96.90	2.35	0.81	5	99.23	2.45	0.81	6
$KFSD_{wei}$	100.00	2.71	0.80	5	97.48	2.13	0.82	4	99.81	2.66	0.80	7
MUOD	96.05	5.84	0.63	7	96.71	6.54	0.61	7	95.18	1.60	0.84	5
$MUOD_{mag}$	95.85	0.50	0.93	3	0.00	2.21	-	-	68.98	0.16	0.80	7
$MUOD_{amp}$	0.59	0.93	0.01	12	96.71	0.62	0.93	1	4.62	0.98	0.08	12
$MUOD_{sha}$	4.94	4.79	0.05	11	0.00	5.62	-	-	83.04	0.50	0.86	4

Focusing on summary indices such as the F-measures, the results in Table S1 show that:

- The proposed type-oriented methods are always among the four best methods. More in detail, $MUOD_{mag}$ is the third best method in presence of magnitude outliers, $MUOD_{amp}$

is the best method in presence of amplitude outliers, which are probably the most challenging outliers to detect, and MUOD_{sha} is the fourth best method in presence of shape outliers. Therefore, one the main novelties of our methods, that is, their type-orientation, it is supported by good and stable results for the considered mixture models.

- MUOD, which is the method obtained by the simultaneous use of MUOD_{mag}, MUOD_{amp} and MUOD_{sha} on a single data set, has a remarkably good performance since it is always among the seven best methods. Clearly, for the considered mixture models, MUOD slightly suffers in terms of false outlier detection percentages since it searches for all type of outliers while all the simulated data sets contains (eventually) only one type of outliers.

- Concerning our competitors: B_{wei} and KFSD_{smo} perform better than MUOD_{mag} in presence of magnitude outliers, however these are methods that entail important computational limitations; the same methods and B_{tri} perform better than MUOD_{sha} in presence of shape outliers, but the remark on the computational limitations still holds; FBAG is the best competitor of MUOD_{amp} in presence amplitude outliers, however it shows poor performances in presence of magnitude and shape outliers.

5.2 Mixture Model 4

In Figure S15 we show a simulated data set obtained using a mixture of models (6), (7), (8) and (9) (mixture model 4) with $\alpha = 0.05$ being the probability that each curve is an outlier and $\frac{1}{3}$ being the probability that each outlier is a magnitude, amplitude and shape outlier, respectively.

For mixture model 4 the comparison among methods is based on 300 generated data sets and is performed as in Table S1. The results are shown in Table S2.

Note that for the type-oriented methods, i.e., OG, MUOD_{mag}, MUOD_{amp} and MUOD_{sha}, the correct outlier detection percentages of Table S2 are difficult to interpret because these methods search for a specific type of outliers. Therefore, to interpret the performances of OG, MUOD_{mag}, MUOD_{amp} and MUOD_{sha} that we observe in Table S2, it is necessary to decompose the outlier detection problem into three parts. This decomposition is presented

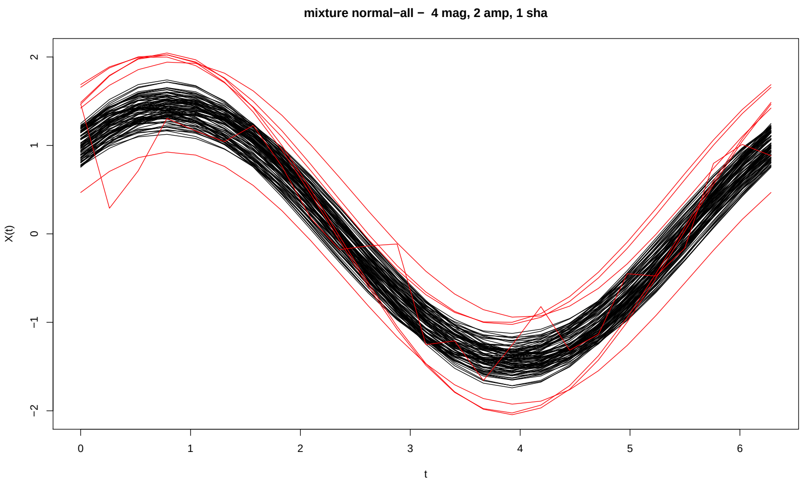

Figure S15: Mixture model 4: magnitude, amplitude and shape outliers.

Table S2: Correct outlier detection percentages (c), false outlier detection percentages (f), F-measures (F) and F-measure-based rankings of the methods (r) in the mixture model 4 which allows simultaneously for magnitude, amplitude and shape outliers.

	$mag-amp-sha$			
	c	f	F	r
B_{tri}	61.81	0.00	0.77	6
B_{wei}	96.21	0.00	0.98	1
FBAG	35.32	0.26	0.50	9
FHDR	42.32	3.00	0.42	11
FBPLOT	34.92	0.00	0.52	8
OG	0.52	0.00	0.02	13
$KFSD_{smo}$	82.67	0.14	0.89	2
$KFSD_{tri}$	99.35	2.34	0.82	4
$KFSD_{wei}$	99.80	2.51	0.81	5
MUOD	97.58	2.01	0.83	3
$MUOD_{mag}$	41.33	0.08	0.57	7
$MUOD_{amp}$	34.01	0.37	0.48	10
$MUOD_{sha}$	30.22	1.61	0.37	12

in Table S3. Note that this table has the same structure of Table S1, but it is associated to a fairly more complex outlier detection problem: in this case the observations and the three types of outliers are generated from a unique mixture model. Clearly, since the non-type-oriented methods search for any type of outliers, their false outlier detection percentages of Table S3 are also difficult to interpret.

Table S3: Decomposed correct outlier detection percentages (c), false outlier detection percentages (f), F-measures (F) and F-measure-based rankings of the methods (r) in mixture model 4 allowing simultaneously for magnitude (*mag*), amplitude (*amp*) and shape (*sha*) outliers.

	mag				*amp*				*sha*			
	c	f	F	r	c	f	F	r	c	f	F	r
B_{tri}	73.08	1.92	0.52	3	21.40	2.84	0.14	9	89.98	1.65	0.63	1
B_{wei}	100.00	3.23	0.52	3	88.60	3.49	0.45	4	99.80	3.27	0.52	4
FBAG	0.77	2.07	0.01	10	98.40	0.42	0.88	2	8.64	1.94	0.08	11
FHDR	8.65	4.94	0.04	9	98.00	3.42	0.49	3	22.00	4.71	0.11	10
FBPLOT	40.19	1.10	0.39	6	1.00	1.79	0.01	11	62.87	0.73	0.61	3
OG	0.00	0.03	-	-	1.60	0.00	0.04	10	0.00	0.03	-	-
$KFSD_{smo}$	100.00	2.66	0.57	2	69.80	3.24	0.39	5	77.60	3.09	0.43	5
$KFSD_{tri}$	100.00	5.64	0.39	6	98.40	5.74	0.37	7	99.61	5.69	0.37	6
$KFSD_{wei}$	100.00	5.84	0.37	8	99.80	5.91	0.36	8	99.61	5.88	0.37	6
MUOD	100.00	5.23	0.40	5	100.00	5.30	0.39	5	92.73	5.39	0.37	6
$MUOD_{mag}$	100.00	0.46	0.88	1	1.80	2.19	0.01	11	20.24	1.87	0.18	9
$MUOD_{amp}$	0.00	2.12	-	-	100.00	0.43	0.89	1	3.93	2.05	0.03	12
$MUOD_{sha}$	1.35	3.10	0.01	10	0.00	3.12	-	-	89.39	1.58	0.63	1

The results in Tables S2 and S3 show that:

- MUOD has a good performance in terms of the F-measures of Table S2. It is the third best method, and it is slightly outperformed by two computational intensive and non-type-oriented methods such as B_{wei} and $KFSD_{smo}$.
- The good performance of MUOD is due to the good and complementary performances of $MUOD_{mag}$, $MUOD_{amp}$ and $MUOD_{sha}$ shown in Table S3: especially $MUOD_{mag}$ and $MUOD_{amp}$ detect very satisfactorily the outliers for which they have been proposed, whereas the performance of $MUOD_{sha}$ is slightly less efficient in terms of its F-measure.

Since mixture model 4 simultaneously allows magnitude, amplitude and shape outliers, it represents a model able to generate data sets that might resemble our real data application. For this reason, we use mixture model 4 to generate a data set having a sample size

comparable to the sample size that we observe in our real data application and we perform outlier detection on it. We generate a data set having 6,000,000 observations and we compare the performances of FBPLOT with the ones of the proposed procedures. We restrict this comparison to these four procedures for computational reasons (see Figure S10).

The simulated data set contains 300,305 outliers (roughly 5%) divided in this way: 99,787 magnitude outliers, 100,166 amplitude outliers and 100,352 shape outliers. In Table S4 we report how many observations are detected by FBPLOT, MUOD$_{mag}$, MUOD$_{amp}$ and MUOD$_{sha}$ as outliers (in the diagonal and in bold), and we also describe the intersection among the outputs of these procedures (outside the diagonal).

Table S4: Information about FBPLOT, MUOD$_{mag}$, MUOD$_{amp}$ and MUOD$_{sha}$ when used on a data set with 6,000,000 observations generated by mixture model 4.

	FBPLOT	MUOD$_{mag}$	MUOD$_{amp}$	MUOD$_{sha}$
FBPLOT	**6934**	1536	65	3785
MUOD$_{mag}$	1536	**118021**	57	4822
MUOD$_{amp}$	65	57	**92336**	135
MUOD$_{sha}$	3785	4822	135	**26708**

According to Table S4, MUOD$_{mag}$ and MUOD$_{amp}$ are the two procedures detecting the most outliers, and their outputs in practice do not overlap. MUOD$_{sha}$ detects less outliers and it shows an appreciable overlap with MUOD$_{mag}$ (18.05% of the observations detected as outliers by MUOD$_{sha}$ are also detected as outliers by MUOD$_{mag}$). FBPLOT is the procedure detecting the least outliers and it shows appreciable overlaps with MUOD$_{mag}$ and MUOD$_{sha}$ (22.15% and 54.59% of the observations detected as outliers by $FBPLOT$ are also detected as outliers by MUOD$_{mag}$ and MUOD$_{sha}$, respectively). A graphical illustration of such results is given in Figure S16.

Tables S5 and S6 replicate Tables S2 and S3, respectively, but now we only consider FBPLOT, MUOD, MUOD$_{mag}$, MUOD$_{amp}$ and MUOD$_{sha}$ as procedures and their performances are evaluated on a unique big data set (with 6,000,000 observations) instead of on many small data sets (300 data sets with 100 observations). In these new tables we omit the F-measure-based rankings.

The results in Tables S5 and S6 when compared to the results in Tables S2 and S3 show that:

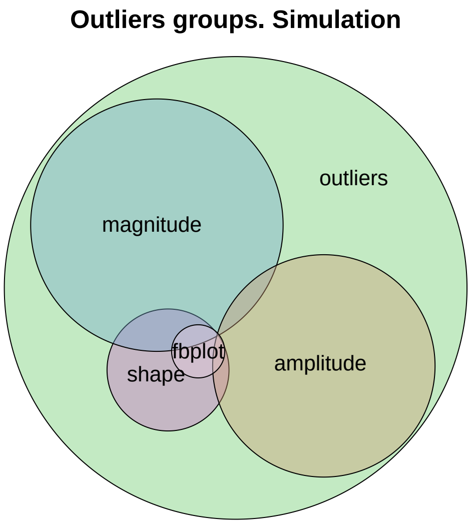

Figure S16: Venn's diagram for outliers and algorithm's detected sets. This plot visually summarizes the results in Tables S4 and S5.

Table S5: Correct outlier detection percentages (c), false outlier detection percentages (f) and F-measures (F) in the mixture model 4 which allows simultaneously for magnitude, amplitude and shape outliers. Performances of FBPLOT, MUOD, $MUOD_{mag}$, $MUOD_{amp}$ and $MUOD_{sha}$ on a data set with 6,000,000 observations.

	$mag - amp - sha$		
	c	f	F
FBPLOT	2.31	0.00	0.05
MUOD	77.28	0.00	0.87
$MUOD_{mag}$	39.30	0.00	0.56
$MUOD_{amp}$	30.75	0.00	0.47
$MUOD_{sha}$	8.89	0.00	0.16

Table S6: Decomposed correct outlier detection percentages (c), false outlier detection percentages (f) and F-measures (F) in mixture model 4 allowing simultaneously for magnitude (*mag*), amplitude (*amp*) and shape (*sha*) outliers. Performances of FBPLOT, MUOD, $MUOD_{mag}$, $MUOD_{amp}$ and $MUOD_{sha}$ on a data set with 6,000,000 observations.

	mag			*amp*			*shape*		
	c	f	F	c	f	F	c	f	F
FBPLOT	0.06	0.12	0.00	0.00	0.12	-	6.85	0.00	0.13
MUOD	100.00	2.24	0.60	91.92	2.37	0.55	40.08	3.25	0.24
$MUOD_{mag}$	100.00	0.31	0.92	0.00	2.00	-	18.17	1.69	0.17
$MUOD_{amp}$	0.00	1.56	-	91.92	0.00	0.96	0.26	1.56	0.00
$MUOD_{sha}$	0.00	0.45	-	0.00	0.45	-	26.61	0.00	0.42

- MUOD improves its performance in terms of F-measures when we compare Table S5 with Table S2. On the contrary, FBPLOT shows an important deterioration of its performance.

- Also MUOD_{mag}, MUOD_{amp} and MUOD_{sha} improve their performances in terms of F-measures when we compare Table S6 with Table S3.

Therefore, MUOD, MUOD_{mag}, MUOD_{amp} and MUOD_{sha} have stable behaviors under mixture model 4 in both small and big sample size scenarios. On the contrary, FBPLOT, experiences a very important deterioration in its outlier detection performance in a big sample size scenario.

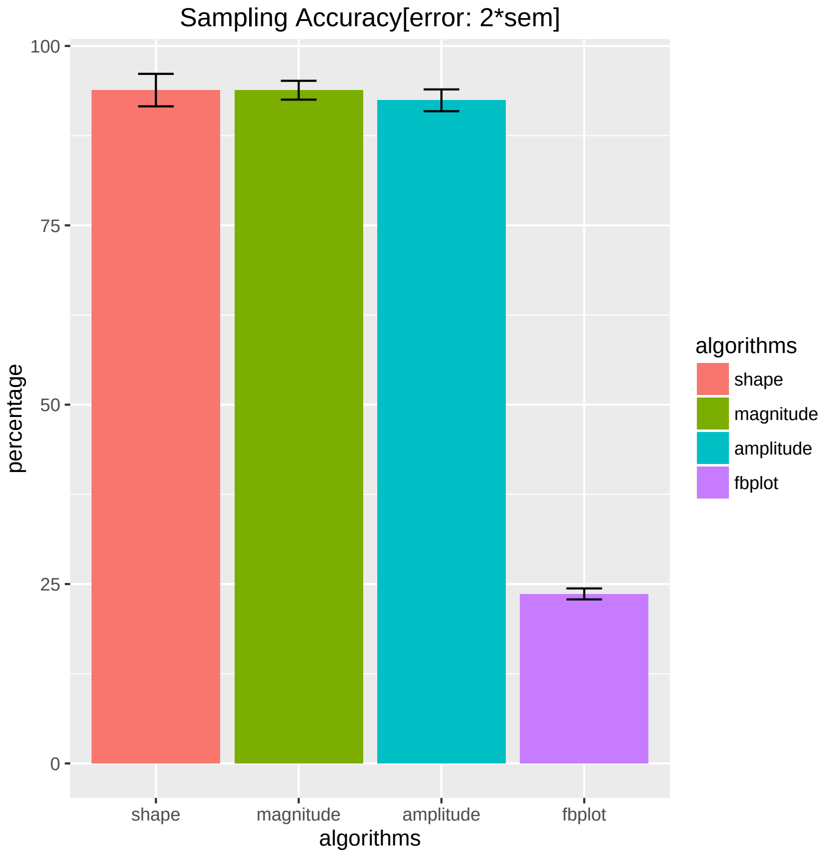

Figure S17: Accuracy for the considered algorithms when sampling. The plot shows average accuracy for 100 simulations using random samples of 100K observations. Each bar shows the accuracy as the intersection ratio between the outliers detected on a sample and the outliers on the total population (6M observations) for an algorithm. The bars show that shape, magnitude and amplitude are not affected when sampling (accuracy $\sim$ 90%) while FBPlot seems to be sensitive to observation samples.

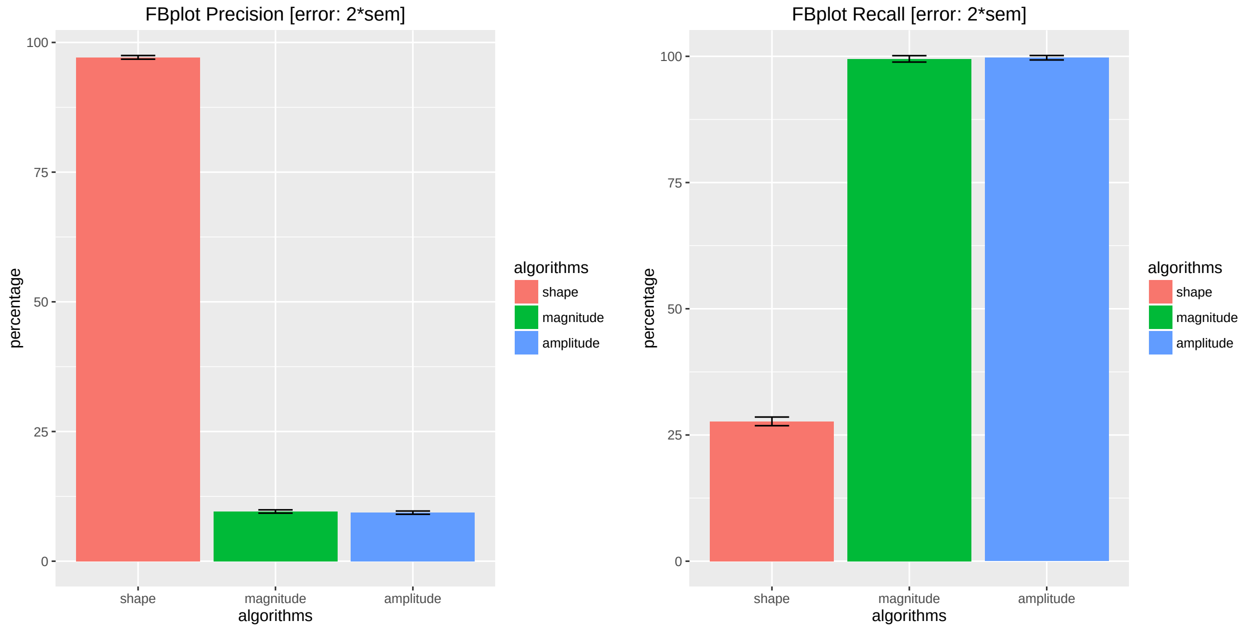

Figure S18: Precision and recall for FBPlot against shape, magnitude and amplitude when sampling. The plot shows average precision and recall for 100 simulations using random samples of 100K observations. Each bar shows that there is a considerable overlap between FBPlot and shape, magnitude and amplitude, yet in different ways. While FBPlot and shape overlap in each sample, FBPlot becomes a small subset of shape. On the other hand, FBPlot overlaps with magnitude and amplitude too, but in this case, magnitude and amplitude become small subsets of FBPlot. To sum up, these plots extend Figures S16 and S17, and show the FBPlot behavior as comparison with MUOD (shape, magnitude, and amplitude) when sampling.

We explore now the behavior of the different algorithms applied over samples of a large population instead of over the whole population. For that, we use the population of 6,000,000 observations described above, and take random samples of 100,000 observations. Figures S17 and S18 present the accuracy, precision, and recall observed when performing this sampling. This experiment shows that the outliers identified by MUOD in the samples matches in a large degree those identified in the whole population. This is not the case with FBPlot.

5.3 Mixture Models 5, 6, and 7

We expand our simulation study considering three additional mixture models that were used in [39] to evaluate OG. We refer to them as mixture model 5, 6 and 7, respectively. Since OG was proposed to expressly detect shape outliers, these mixture models generate different types of shape outliers, which are also the type of outliers that until now our procedures seem to have the most problems to detect.

- In mixture model 5 the main model is given by

$$X(t) = 30t(1-t)^{3/2} + \epsilon(t),$$

where $t \in [0, 1]$ and $\epsilon(t)$ is a Gaussian process with zero mean and covariance function $\gamma(s, t) = 0.3 \exp\{-|s-t|/0.3\}$, while the contamination model is given by

$$X(t) = 30t^{3/2}(1-t) + \epsilon(t).$$

- In mixture model 6 the main model is given by

$$X(t) = 4t + \epsilon(t), \tag{11}$$

where $t \in [0, 1]$ and $\epsilon(t)$ is a Gaussian process with zero mean and covariance function $\gamma(s, t) = \exp\{-|s-t|\}$, while the contamination model is given by

$$X(t) = 4t + (-1)^u 1.8 + \frac{1}{\sqrt{2\pi 0.01}} \exp\left\{-(t-\mu)^2/0.02\right\} + \epsilon(t),$$

where u follows a Bernoulli distribution with probability $1/2$ and μ is uniformly distributed in $[0.25, 0.75]$.

- In mixture model 7 the main model is given by (11), with t and $\epsilon(t)$ defined as in mixture model 6, while the contamination model is given by

$$X(t) = 4t + 2\sin(4(t + \mu)\pi) + \epsilon(t),$$

with μ defined as in mixture model 6.

In Figure S19 we show three simulated data sets obtained using mixture models 5, 6 and 7 with $d = 50$ equidistant points between 0 and 1, $n = 100$ and $\alpha = 0.05$ being the probability that each curve is an outlier in each model.

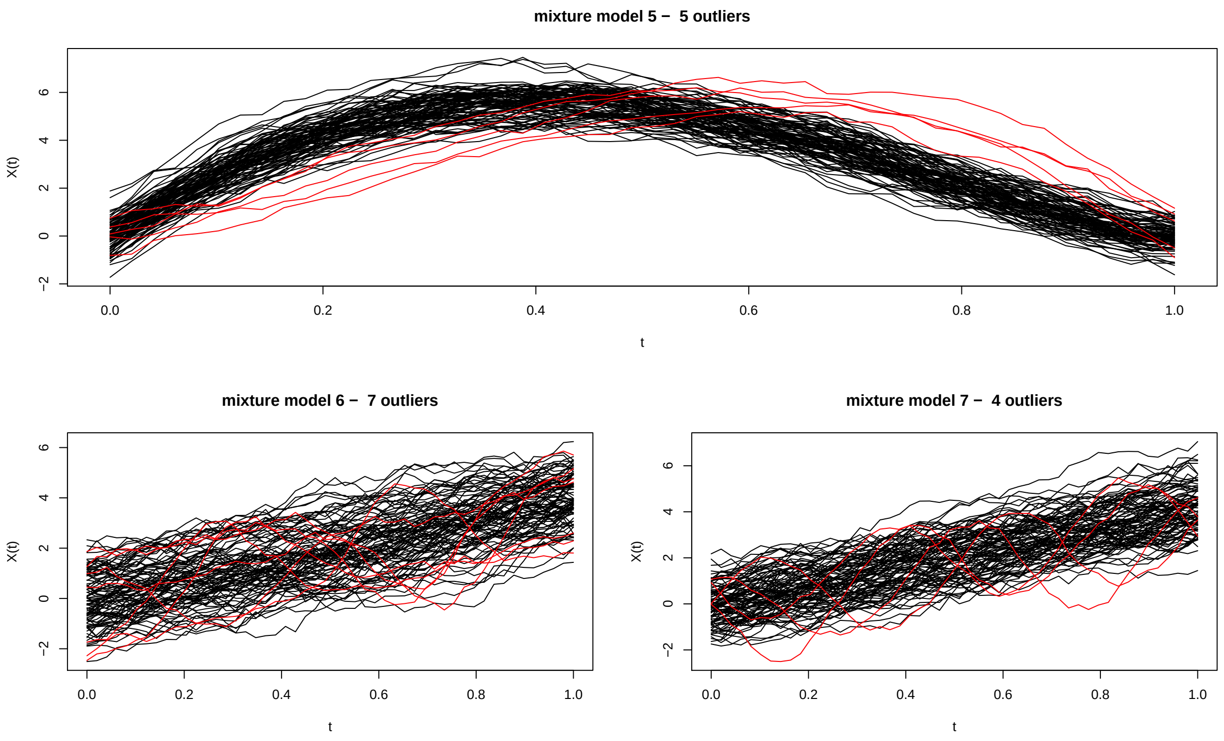

Figure S19: Mixture models 5 (top), 6 (bottom left) and 7 (bottom right).

The comparison among methods for mixture models 5, 6 and 7 is performed similarly as for the previous four models, and it is reported in Table S7.

The results in Table S7 show that:

Table S7: Correct outlier detection percentages (c), false outlier detection percentages (f), F-measures (F) and F-measure-based rankings of the methods (r) in mixture models 5, 6 and 7.

	mix mod 5				*mix mod 6*				*mix mod 7*			
	c	f	F	r	c	f	F	r	c	f	F	r
B_{tri}	48.23	0.03	0.65	10	26.20	0.59	0.38	8	23.55	0.79	0.34	8
B_{wei}	88.08	0.41	0.90	2	30.59	0.71	0.43	7	23.55	0.76	0.35	7
FBAG	99.63	6.70	0.63	11	36.14	7.00	0.27	9	8.58	7.86	0.06	10
FHDR	65.74	1.55	0.68	8	23.71	3.97	0.24	10	5.59	4.97	0.06	10
FBPLOT	26.44	0.01	0.41	13	0.19	0.00	0.00	13	0.40	0.02	0.00	13
OG	97.95	2.31	0.82	4	98.85	3.36	0.76	1	100.00	3.92	0.73	1
$KFSD_{smo}$	84.92	0.55	0.87	3	49.52	3.05	0.48	5	45.71	3.67	0.43	5
$KFSD_{tri}$	98.14	4.36	0.71	7	79.54	6.29	0.54	4	82.04	6.33	0.55	3
$KFSD_{wei}$	99.26	5.27	0.68	8	86.81	7.02	0.56	3	88.22	7.22	0.54	4
MUOD	95.53	3.42	0.75	5	67.30	6.91	0.46	6	67.66	7.47	0.44	5
$MUOD_{mag}$	47.49	1.65	0.53	12	9.37	3.17	0.11	12	1.00	3.92	0.01	12
$MUOD_{amp}$	72.63	1.60	0.72	6	14.53	3.49	0.17	11	6.19	3.75	0.07	9
$MUOD_{sha}$	92.74	0.53	0.92	1	60.04	2.24	0.60	2	66.07	2.15	0.64	2

- As expected, for the considered mixture models, $MUOD_{sha}$ is the appropriate method among the proposed methods. Moreover, it is always among the two best methods for these mixture models generating shape outliers.
- MUOD has also a good performance since it is always among the six best methods and this is due to the fact that both $MUOD_{mag}$ and $MUOD_{amp}$ do not detect many false outliers.
- The best competitors for $MUOD_{sha}$ and MUOD are B_{wei}, the KFSD-based techniques and OG. For B_{wei} and the KFSD-based techniques hold the remarks made above. For OG, its good performances in mixture models 5, 6 and 7 contrast with its poor performances in mixture models 1, 2 , 3 and 4, and therefore OG does not represent a real competitor for our proposals.

After comparing our methods with a wide set of competitors in standard FDA simulated scenarios and showing their good performances, in the next section we present some supplementary details on our real data application. As for the 6,000,000 observations run of mixture model 4, also in this case we restrict the analysis to the proposed procedures and FBPLOT.

6 Outlier Detection in our Real Data Application

In this section we present some remarks on the outlier detection in our real data application. Recall that our real data set describes 21 features of 5,619,786 users of Google+ and that, after discarding 2 features, we treat it as a functional data set.

Table S9 replicates Table S4: first, we report how many observations are detected by FBPLOT, MUOD_{mag}, MUOD_{amp} and MUOD_{sha} as outliers (in the diagonal and in bold); second, we describe the interaction among the outputs of these procedures (outside the diagonal).

Table S8: Dataset variables description.

Variable	Description
id	The user identifier in Google+
NumActivities	The total number of activities (posts) of a user
NumAttachments	Total number of attachments included in the posts made by the user
NumPlusOnes	The total number of '+1' the user's posts have received
NumReplies	The total number of replies the user's posts have received
NumReshares	The total number of reshares the user's posts have been done
NumFriends	The number of friends the user has
NumFollowers	The number of followers the user has
NumFields	Number of fields the user made public in the profile
PercentageBidirectional	Percentage of friends which are following each other
accountAge	Number of days since first time we say the account active (i.e., first post), measured from the data collection instant
accountRecency	Number of days since last post, measured from the data collection instant
gender	The gender declared by the user
job	The job declared by the user
numVideos	The number of videos the user has published
numPhotos	The number of photos the user has published
numAlbums	The number of albums the user has
numArticles	The number of articles the user has published
numHangouts	Number of hangouts made by the user
numEvents	Number of events the user participated in
numWithGeo	Number of posts with geographical information
pageRank	The topological pageRank value of the user considering the whole network (unweighted)

Note that according to FBPLOT, MUOD_{mag}, MUOD_{amp} and MUOD_{sha} the 0.29%, 0.11%, 0.11% and 5.38% of the observations are outliers, respectively. Moreover,

- All the observations that are detected as outliers by FBPLOT, except 18, are also

Table S9: Information about FBPLOT, MUOD_{mag}, MUOD_{amp} and MUOD_{sha} when used on our real data application.

	FBPLOT	MUOD_{mag}	MUOD_{amp}	MUOD_{sha}
FBPLOT	**16140**	4366	6074	16122
MUOD_{mag}	4366	**6103**	4036	6103
MUOD_{amp}	6074	4036	**6178**	6178
MUOD_{sha}	16122	6103	6178	**302345**

detected as outliers by MUOD_{sha}.

- All the observations that are detected as outliers by MUOD_{mag} and MUOD_{amp} are also detected as outliers by MUOD_{sha}.

Therefore, according to the proposed procedures in our real data application if an observation is detected as an outlier, it is at least a shape outlier. Due to this result, let us define the following types of observations:

1. *no MUOD*: observations that are normal according to MUOD_{mag}, MUOD_{amp} and MUOD_{sha}.

2. *only sha*: observations that are detected as outliers by MUOD_{sha} and are normal observations according to MUOD_{mag} and MUOD_{amp}.

3. *only mag+sha*: observations that are detected as outliers by MUOD_{sha} and MUOD_{mag} and are normal observations according MUOD_{amp}.

4. *only amp+sha*: observations that are detected as outliers by MUOD_{sha} and MUOD_{amp} and are normal observations according MUOD_{amp}.

5. *mag+amp+sha*: observations that are detected as outliers by MUOD_{sha}, MUOD_{mag} and MUOD_{amp}.

Note that the all the observations in our real data set belong exclusively to one of the classes of above. In Table S10 we describe how the observations are distributed in these classes (in absolute and relative terms).

In Figure S20 we represent the observations as functional data†:

†We use a logarithmic transformation that avoids the argument of the logarithm to be non-positive.

Table S10: Distribution of the observations of our real data application in the classes *no MUOD*, *only sha*, *only mag+sha*, *only amp+sha* and *mag+amp+sha*. Absolute (*abs*) and relative (*rel*) frequencies.

	abs	*rel*
no MUOD	5317441	0.9462
only sha	294100	0.0523
only mag+sha	2067	0.0004
only amp+sha	2142	0.0004
mag+amp+sha	4036	0.0007
totals	5619786	1.0000

a. a 1% of the 294100 *only sha* observations (top left).

b. the 2067 *only mag+sha* observations (top right).

c. the 2142 *only amp+sha* observations (bottom left).

d. the 4036 *only mag+amp+sha* observations (bottom right).

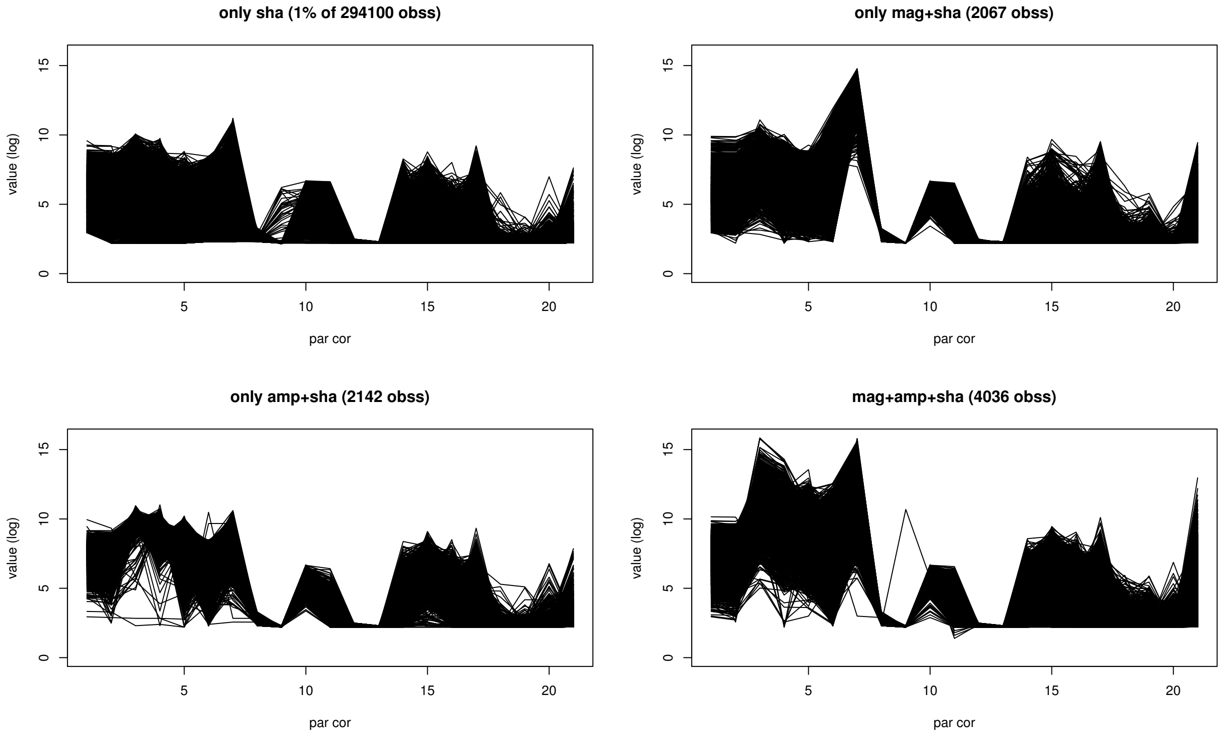

Figure S20: 1% *only sha*, *only mag+sha*, *only amp+sha* and *mag+amp+sha* observations (from top left, in clockwise order).

Observing Figure S20 we notice that the four classes of observations detected as outliers by the proposed procedures would make functional data sets different among them. Such a result seems to justify the detection of four different types of outliers. Moreover, thanks to Figure S21, where we represent 5000 *no MUOD* observations as functional data, it is possible to appreciate another important fact, i.e., there are differences between the observations detected as outliers and the observations that are normal according to the proposed procedures. For more qualitative details on these differences, see the main article.

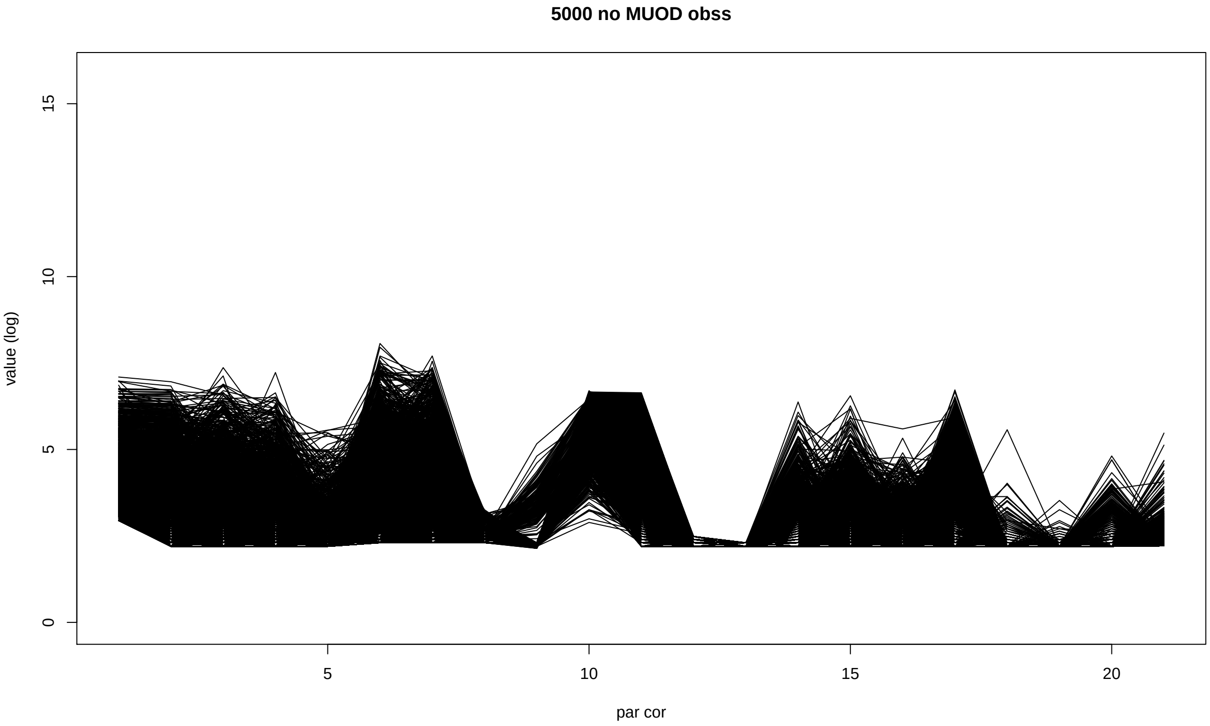

Figure S21: 5000 *no MUOD* observations.

Once defined the classes *no MUOD*, *only sha*, *only mag+sha*, *only amp+sha* and *mag+amp+sha*, and before closing this section, in Tables S11 and S12 we show their distributions against two classes related with the FBPLOT procedure: *no FBPLOT* and *yes FBPLOT*, the observations that are normal and outlying according to FBPLOT, respectively.

According to Tables S11 and S12, FBPLOT in practice does not detect as outliers those observations that were not detected as outliers by the proposed procedures. Moreover, the 99.81% and 97.52% of *only amp+sha* and *mag+amp+sha* observations are outlying

Table S11: Distribution of the observations of our real data application in the classes *no MUOD*, *only sha*, *only mag+sha*, *only amp+sha* and *mag+amp+sha* against the classes *no FBPLOT* and *yes FBPLOT*. Absolute frequencies.

	no FBPLOT	*yes FBPLOT*	*totals*
no MUOD	5317423	18	5317441
only sha	284482	9618	294100
only mag+sha	1637	430	2067
only amp+sha	4	2138	2142
mag+amp+sha	100	3936	4036
totals	5603646	16140	5619786

Table S12: Distribution of the observations of our real data application in the classes*no MUOD*, *only sha*, *only mag+sha*, *only amp+sha* and *mag+amp+sha* against the classes *no FBPLOT* and *yes FBPLOT*. Relative frequencies inside the classes *no MUOD*, *only sha*, *only mag+sha*, *only amp+sha* and *mag+amp+sha*.

	no FBPLOT	*yes FBPLOT*	totals
no MUOD	1.0000	0.0000	1
only sha	0.9673	0.0327	1
only mag+sha	0.7920	0.2080	1
only amp+sha	0.0019	0.9981	1
mag+amp+sha	0.0248	0.9752	1

also according to FBPLOT, respectively. The degree of concordance between the proposed procedures and FBPLOT falls to 20.80% and 3.27% in the classes *only mag+sha* and *only sha*, respectively. Therefore, the main differences between proposed procedures and FBPLOT can be observed in the classes *only mag+sha* and especially *only sha*.

In Figures S22-S24 we represent as functional data:

a. Figure S22: a 1% of the 284482 *(only sha, no FBPLOT)* observations (left) and a 25% of the *(only sha, yes FBPLOT)* observations (right).

b. Figure S23: the 1637 *(only mag+sha, no FBPLOT)* observations (left) and the 430 *(only mag+sha, yes FBPLOT)* observations (right).

c. Figure S24: the 100 *(mag+amp+sha, no FBPLOT)* observations (left) and the 3936 *(mag+amp+sha, yes FBPLOT)* observations (right).

In Figures S22-S24 we also draw the upper bound of the non-outlying region defined by FBPLOT.

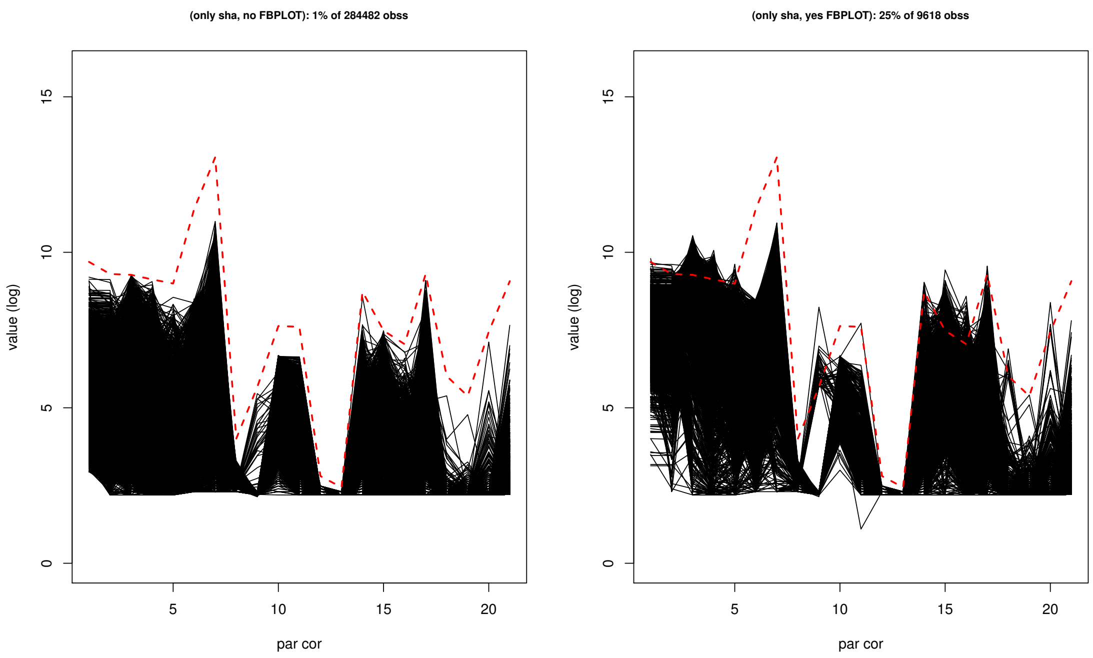

Figure S22: 1% *(only sha, no FBPLOT)* observations (left); 25% *(only sha, yes FBPLOT)* observations (right). Dashed line in both figures: upper bound of the non-outlying region defined by FBPLOT.

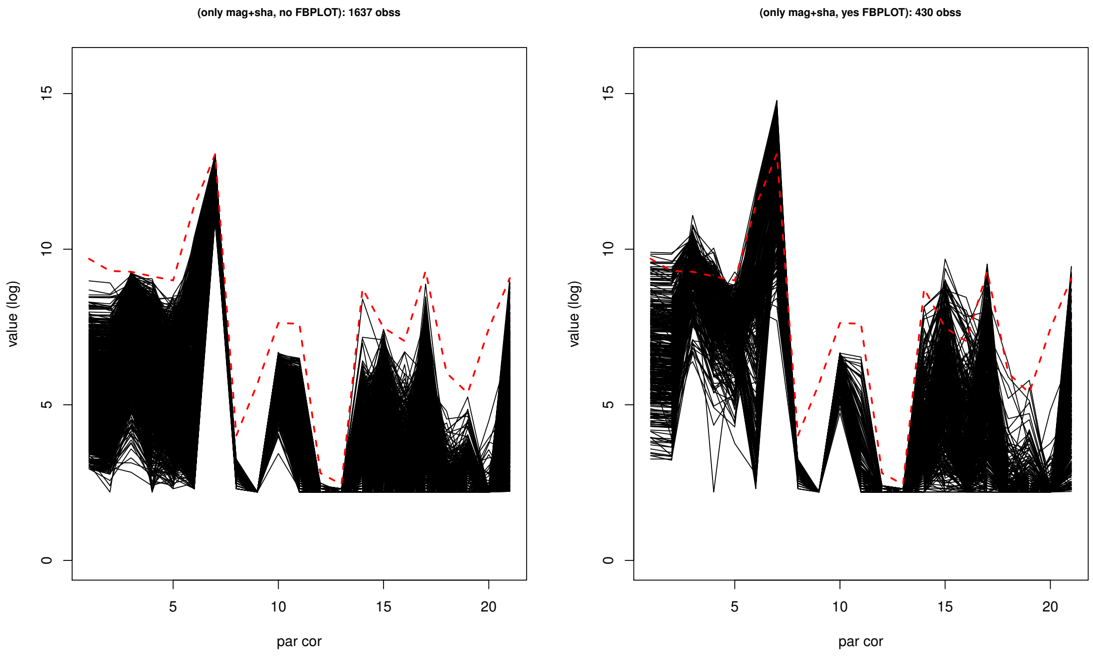

Figure S23: *(only mag+sha, no FBPLOT)* observations (left); *(only mag+sha, yes FBPLOT)* observations (right). Dashed line in both figures: upper bound of the non-outlying region defined by *FBPLOT*.

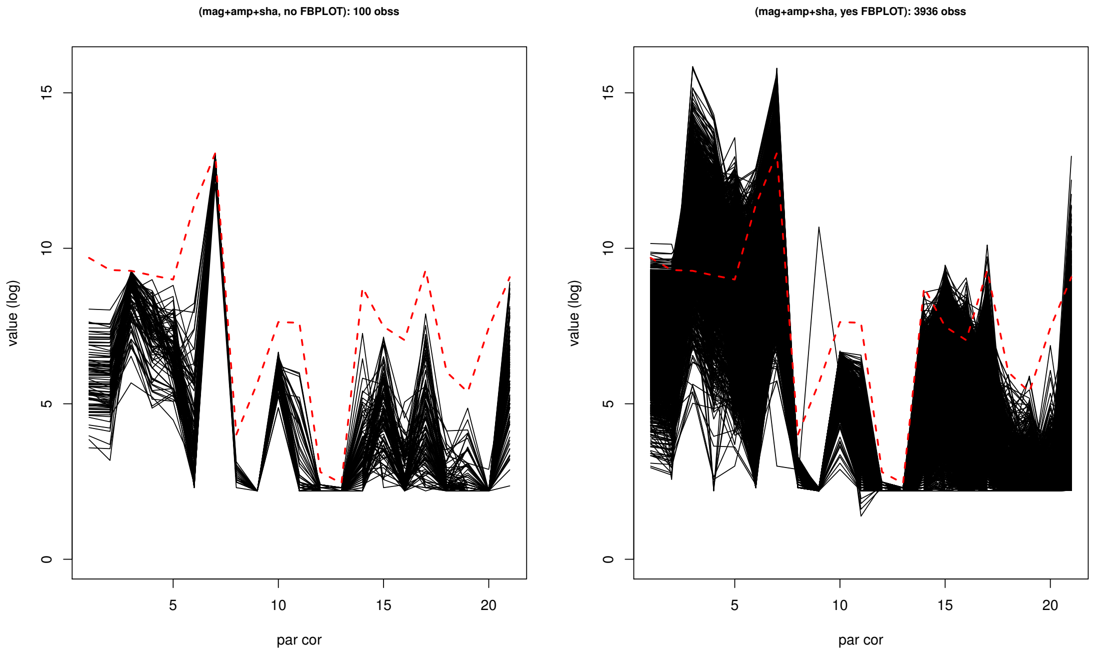

Figure S24: *(mag+amp+sha, no FBPLOT)* observations (left); *(mag+amp+sha, yes FBPLOT)* observations (right). Dashed line in both figures: upper bound of the non-outlying region defined by FBPLOT.

Observing Figures S22-S24 we notice that the proposed procedures detect as outliers more observations than FBPLOT, especially as *only sha* outliers. These observations, when compared to some of the observations that are normal according to the proposed procedures (Figure S21), show a behavior that seems to justify their detection as outliers. For more qualitative details on the differences between MUOD and FBPLOT, see the main article.

References

[1] Jin, L., Chen, Y., Wang, T., Hui, P. & Vasilakos, A. V. Understanding user behavior in online social networks: A survey. *IEEE Communications Magazine* **51**, 144–150 (2013).

[2] de Arruda, G. F. *et al.* Role of centrality for the identification of influential spreaders in complex networks. *Physical Review E* **90**, 032812 (2014).

[3] Kitsak, M. *et al.* Identification of influential spreaders in complex networks. *Nature physics* **6**, 888–893 (2010).

[4] Morone, F. & Makse, H. A. Influence maximization in complex networks through optimal percolation. *Nature* **524**, 65–68 (2015).

[5] Kempe, D., Kleinberg, J. M. & Tardos, É. Maximizing the spread of influence through a social network. *Theory of Computing* **11**, 105–147 (2015). URL `https://doi.org/10.4086/toc.2015.v011a004`.

[6] Domingos, P. M. & Richardson, M. Mining the network value of customers. In *Proceedings of the seventh ACM SIGKDD international conference on Knowledge discovery and data mining, San Francisco, CA, USA, August 26-29, 2001*, 57–66 (2001). URL `http://portal.acm.org/citation.cfm?id=502512.502525`.

[7] D'Agostino, G., D'Antonio, F., De Nicola, A. & Tucci, S. Interests diffusion in social networks. *Physica A: Statistical Mechanics and its Applications* **436**, 443–461 (2015).

[8] Bakshy, E., Hofman, J. M., Mason, W. A. & Watts, D. J. Identifying influencers on twitter. In *Fourth ACM International Conference on Web Seach and Data Mining (WSDM)* (2011).

[9] Basaras, P., Katsaros, D. & Tassiulas, L. Detecting influential spreaders in complex, dynamic networks. *Computer* **46**, 24–29 (2013).

[10] Cha, M., Haddadi, H., Benevenuto, F. & Gummadi, P. K. Measuring user influence in twitter: The million follower fallacy. *ICWSM* **10**, 30 (2010).

[11] Simmie, D., Vigliotti, M. G. & Hankin, C. Ranking twitter influence by combining network centrality and influence observables in an evolutionary model. *Journal of Complex Networks* **2**, 495–517 (2014).

[12] Hubert, M., Rousseeuw, P. J. & Segaert, P. Multivariate functional outlier detection. *Statistical Methods and Applications* **24**, 177–202 (2015).

[13] Febrero, M., Galeano, P. & González-Manteiga, W. Outlier detection in functional data by depth measures, with application to identify abnormal nox levels. *Environmetrics* **19**, 331–345 (2008).

[14] Louail, T. *et al.* From mobile phone data to the spatial structure of cities. *Scientific Reports* (2014).

[15] Gonzalez, R., Rumín, R. C., Motamedi, R., Rejaie, R. & Cuevas, Á. Assessing the evolution of google+ in its first two years. *IEEE/ACM Trans. Netw.* **24**, 1813–1826 (2016). URL http://dx.doi.org/10.1109/TNET.2015.2433792.

[16] Guo, L., Tan, E., Chen, S., Zhang, X. & Zhao, Y. E. Analyzing patterns of user content generation in online social networks. In *Proceedings of the 15th ACM SIGKDD international conference on Knowledge discovery and data mining*, 369–378 (ACM, 2009).

[17] Leskovec, J., McGlohon, M., Faloutsos, C., Glance, N. & Hurst, M. Patterns of cascading behavior in large blog graphs. In *Proceedings of the 2007 SIAM international conference on data mining*, 551–556 (SIAM, 2007).

[18] Sun, Y. & Genton, M. G. Functional boxplots. *Journal of Computational and Graphical Statistics* **20**, 316–334 (2011).

[19] López-Pintado, S. & Romo, J. On the concept of depth for functional data. *Journal of the American Statistical Association* **104**, 718–734 (2009).

[20] Pastor-Satorras, R. & Vespignani, A. Epidemic spreading in scale-free networks. *Physical review letters* **86**, 3200 (2001).

[21] Cohen, R., Erez, K., Ben-Avraham, D. & Havlin, S. Breakdown of the internet under intentional attack. *Physical review letters* **86**, 3682 (2001).

[22] Lazar, N. *The statistical analysis of functional MRI data* (Springer Science & Business Media, 2008).

[23] Lindquist, M. A. The statistical analysis of fmri data. *Statistical Science* 439–464 (2008).

[24] Monti, M. M. Statistical analysis of fmri time-series: a critical review of the glm approach. *Frontiers in human neuroscience* **5** (2011).

[25] Poline, J.-B. & Brett, M. The general linear model and fmri: does love last forever? *Neuroimage* **62**, 871–880 (2012).

[26] Chen, Y., Dang, X., Peng, H. & Bart, H. L. Outlier detection with the kernelized spatial depth function. *IEEE Transactions on Pattern Analysis and Machine Intelligence* **31**, 288–305 (2009).

[27] Zeng, Y. *et al.* Aberrant gene expression in humans. *PLoS genetics* **11**, e1004942 (2015).

[28] Reimann, C. & Filzmoser, P. Normal and lognormal data distribution in geochemistry: death of a myth. consequences for the statistical treatment of geochemical and environmental data. *Environmental geology* **39**, 1001–1014 (2000).

[29] Templ, M., Filzmoser, P. & Reimann, C. Cluster analysis applied to regional geochemical data: problems and possibilities. *Applied Geochemistry* **23**, 2198–2213 (2008).

[30] Wegman, E. J. Hyperdimensional data analysis using parallel coordinates. *Journal of American Statistical Association* **85**, 664–675 (1990).

[31] Ramsay, J. O. & Silverman, B. W. *Functional Data Analysis* (Springer, New York, 2005).

[32] Ferraty, F. & Vieu, P. *Nonparametric Functional Data Analysis : Theory and Practice* (Springer, New York, 2006).

[33] Horváth, L. & Kokoszka, P. *Inference for Functional Data With Applications* (Springer, New York, 2012).

[34] Cuevas, A. A partial overview of the theory of statistics with functional data. *Journal of Statistical Planning and Inference* **147**, 1–23 (2014).

[35] Bapna, R. & Umyarov, A. Do your online friends make you pay? a randomized field experiment on peer influence in online social networks. *Management Science* **61**, 1902–1920 (2015).

[36] Liu, S. *et al.* Identifying effective influencers based on trust for electronic word-of-mouth marketing: A domain-aware approach. *Information Sciences* **306**, 34–52 (2015).

[37] Sguera, C., Galeano, P. & Lillo, R. Spatial depth-based classification for functional data. *TEST* **23**, 725–750 (2014).

[38] Sguera, C., Galeano, P. & Lillo, R. E. Functional outlier detection by a local depth with application to nox levels. *Stochastic environmental research and risk assessment* **30**, 1115–1130 (2016).

[39] Arribas-Gil, A. & Romo, J. Shape outlier detection and visualization for functional data: the outliergram. *Biostatistics* **15**, 603–619 (2014).

[40] Cuevas, A., Febrero, M. & Fraiman, R. On the use of the bootstrap for estimating functions with functional data. *Computational Statistics and Data Analysis* **51**, 1063–1074 (2006).

[41] Hyndman, R. J. & Shang, H. L. Rainbow plots, bagplots, and boxplots for functional data. *Journal of Computational and Graphical Statistics* **19**, 29–45 (2010).

[42] Tukey, J. W. Mathematics and the picturing of data. In *Proceedings of the International Congress of Mathematicians*, vol. 2, 523–531 (1975).

[43] López-Pintado, S. & Romo, J. A half-region depth for functional data. *Computational Statistics and Data Analysis* **55**, 1679–1695 (2011).

[44] Rijsbergen, C. J. V. *Information Retrieval* (Butterworth-Heinemann, Newton, MA, USA, 1979), 2nd edn.
